# Nanoparticle-assembled bioadhesive coacervate coating with prolonged gastrointestinal retention for inflammatory bowel disease therapy

**DOI:** 10.1038/s41467-021-27463-6

**Published:** 2021-12-09

**Authors:** Pengchao Zhao, Xianfeng Xia, Xiayi Xu, Kevin Kai Chung Leung, Aliza Rai, Yingrui Deng, Boguang Yang, Huasheng Lai, Xin Peng, Peng Shi, Honglu Zhang, Philip Wai Yan Chiu, Liming Bian

**Affiliations:** 1 Department of Biomedical Engineering, The Chinese University of Hong Kong, Hong Kong, 999077 China; 2 Department of Endoscopy, State Key Laboratory of Oncology in South China, Collaborative Innovation Center for Cancer Medicine, Sun Yat-sen University Cancer Center, Guangzhou, 510000 China; 3 Chow Yuk Ho Technology Centre for Innovative Medicine, The Chinese University of Hong Kong, Hong Kong, 999077 China; 4 Department of Surgery, Institute of Digestive Disease, State Key Laboratory of Digestive Disease, The Chinese University of Hong Kong, Hong Kong, 999077 China; 5 School of Biomedical Sciences and Engineering, Guangzhou International Campus, South China University of Technology, Guangzhou, 511442 China; 6 National Engineering Research Center for Tissue Restoration and Reconstruction, South China University of Technology, Guangzhou, 510006 China

**Keywords:** Drug delivery, Ulcerative colitis, Self-assembly, Biomedical materials, Biomedical materials

## Abstract

A key challenge for the effective treatment of gastrointestinal diseases including inflammatory bowel disease is to develop an orally administered drug delivery system capable of prolonged retention in the gastrointestinal tract. Herein we report a bioadhesive liquid coacervate based on hydrogen bonding-driven nanoparticle assembly. Free from electrostatic interactions, our fluid nanoparticle-assembled coacervate demonstrates significant pH- and salt-independent structural stability and forms a physically adhesive coating on a large surface area of intestinal tract with an extended residence time of more than 2 days to mediate the sustained release of preloaded water-soluble small molecule drugs in vivo. The orally administered drug-laden nanoparticle-assembled coacervate significantly mitigates the symptoms of inflammatory bowel disease, restores the diversity of gut microbiota, reduces systemic drug exposure, and improves the therapeutic efficacy in a rat acute colitis model compared with the oral administration of the same amount of drug in solution form. We suggest that the nanoparticle-assembled coacervate provides a promising drug delivery platform for management and treatment of numerous gastrointestinal diseases where controlled drug release with extended residence time is desired.

## Abstract (web summary)

The development of a drug delivery system capable of prolonged retention in the gastrointestinal tract remains a clinical challenge. Here the authors present a bio-adhesive liquid coacervate coating on the intestinal tract that acts as a flowable drug carrier, mediates the sustained release of diverse drugs, and potentially enhances therapeutic efficacy against gastrointestinal diseases.

## Introduction

Inflammatory bowel disease (IBD) including Crohn’s disease (CD) and ulcerative colitis (UC) causes chronic relapsing inflammation of the gastrointestinal (GI) tract^1^. IBD significantly increases the risk of developing colorectal cancer and imposes a lifelong healthcare burden on millions of patients worldwide^2–4^. Oral dosage formulations of drugs such as corticosteroids are the most desirable delivery systems available to treat IBD and are vastly superior to enema and subcutaneous and intravenous injection in terms of patient compliance^5^. However, many patients require lifelong administration of drugs to control IBD symptoms; therefore, reducing the severity of side effects attributable to systemic drug exposure resulting from frequent oral dosing via conventional delivery systems, such as enteric-coated tablets and capsules, is essential^6^.

Although many forms of oral drug delivery vehicles have been developed, the insufficient residence time within the intestinal tract and the complex design of delivery vehicles limit their widespread clinical applications^7–10^. Endowing oral drug delivery vehicles with wet bioadhesion may prolong intestinal residence time. However, solid adhesives with high adhesion energy on wet tissues^11–14^ may not be suitable as orally administered intestine-coating drug delivery platforms. For example, we recently reported a bioadhesive hydrogel, which can adhere to gastric ulcer sites upon in situ gelation^15^. However, such adhesive hydrogel cannot form the extensive and uniform adhesive coating layer on the convoluted intestinal tract surfaces due to its solid nature after rapid gelation.

**Fig. 1:**
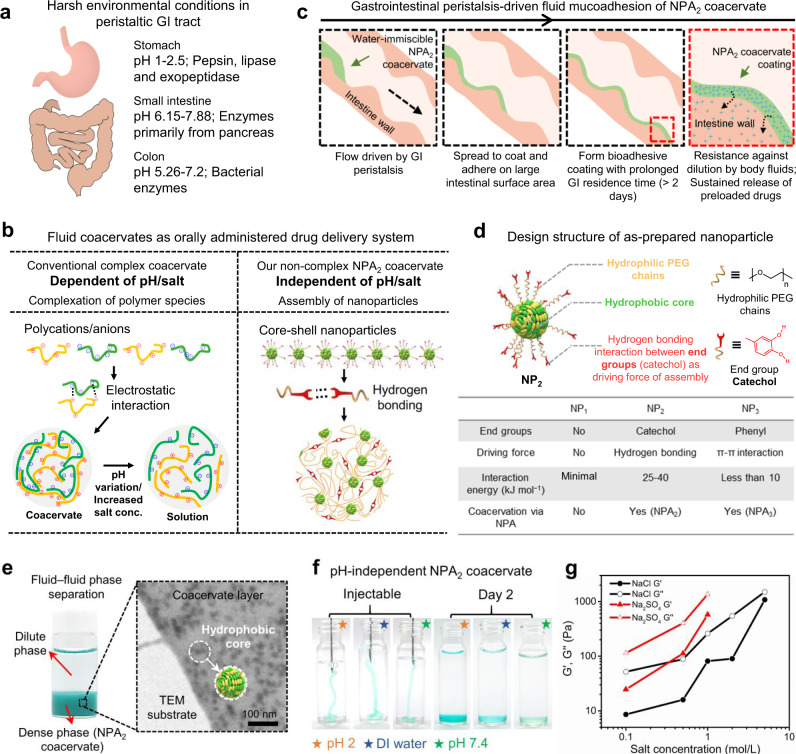
Core-shell nanoparticles assemble into noncomplex coacervate to adapt to the harsh gastrointestinal (GI) tract environment. **a** Schematic illustration of harsh environmental conditions in the peristaltic GI tract. **b** Our noncomplex nanoparticle-assembled (NPA) coacervate compared with the conventional pH- and salt-dependent complex coacervate stabilized by electrostatic interactions between polyanions and polycations. **c** Driven by the gastrointestinal peristalsis, fluid NPA coacervate can effectively spread to coat and adhere on the large intestinal surface area via catechol-mediated wet bioadhesion. **d** The structure of as-prepared core-shell nanoparticles for fabricating NPA coacervates contains a hydrophobic core, hydrophilic PEG chains as the shell, and end groups of the PEG chains that provide physical interactions to induce nanoparticle assembly. The capacities of different nanoparticles (NP_1_, NP_2_, and NP_3_) to form coacervates via nanoparticle assembly were examined. **e** The formation of NPA_2_ coacervate (stained by Fast Green FCF) via NP_2_ nanoparticle assembly can be visualized by fluid–fluid phase separation and further observed by TEM. **f** The liquid-like (G′ < G″) NPA_2_ coacervate (stained by Fast Green FCF) can be injected through a 21 G needle and remained stable in buffers with a wide range of pH after 2 days. **g** Noncomplex NPA_2_ coacervate showed a salting-out effect, confirming that the formation of NPA coacervates should be attributed to hydrogen bonding-induced nanoparticle assembly rather than electrostatic interactions. Source data are provided as a Source Data file for Fig. 1g.

Distinct from the solid bioadhesives, conventional complex coacervates are polymer-concentrated and water-immiscible liquids formed by fluid–fluid phase separation of complexed polyanions and polycations and represent an exciting class of drug delivery vehicles^16–18^. However, the complex coacervation process is driven by electrostatic attraction between polyanions and polycations within narrow pH/salt ranges^19^. Considering that the GI tract routinely undergoes substantial changes in motility, fluid content, and acidity (from pH 1.5 in the stomach to pH 6.15–7.88 in intestines)^5,20^, conventional complex coacervates can be easily disrupted by pH/salt variations (Fig. 1a, b)^21–23^. One recent groundbreaking work reported the precise engineering of low molecular weight sucralfate into complex coacervate to increase the residence time in intestines to several hours^24^. Despite these advances, the limited choices of polyelectrolytes species for preparing complex coacervates that can adapt to the harsh in vivo environment, together with the insufficient intestinal residence time, greatly hamper the development of fluid coacervates as the orally administrated drug delivery systems.

Here we report a universal strategy to prepare water-immiscible, bioadhesive, and noncomplex liquid coacervates derived from bidentate hydrogen bonding-driven self-assembly of nanoparticles as an orally administered intestinal-coating formulation (Fig. 1b and Supplementary Fig. 1). Driven by the gastrointestinal peristalsis, the nanoparticle-assembled fluid coacervate (named NPA coacervate hereafter) can effectively spread to coat and adhere on the large intestinal surface area with a prolonged residence time of more than 2 days to mediate the sustained release of loaded drugs (Fig. 1c). Using the water-soluble dexamethasone sodium phosphate (Dex-P) as a model small molecule drug, we demonstrated that the oral administration of drug-laden NPA coacervate significantly enhanced the IBD therapeutic outcomes, improved the richness and diversity of gut microbiota, and reduced the systemic drug exposure compared with the treatment with Dex-P aqueous solution in a rat model of dextran sulfate sodium (DSS)-induced acute colitis—one of the most widely used models due to its shared clinical and histopathological characteristics with human IBD^25^ and its validation as a relevant model to treat human diseases^26^.

## Results and discussion

### The design of noncomplex NPA coacervates via nanoparticle assembly

Nanoparticles required for fabricating NPA coacervates contain a hydrophobic core and end-functionalized (e.g., catechol) hydrophilic polyethylene glycol (PEG) chains as the shell (Fig. 1d). In this study, MALDI-TOF mass spectrometry was used to confirm the successful synthesis of the hydrophobic core (Supplementary Fig. 2). The molecular weight distribution of the hydrophilic PEG chains was further determined by gel permeation chromatography (GPC, Supplementary Fig. 2). Dynamic light scattering (DLS) analysis confirmed the formation of the as-prepared NP_1_, NP_2_, and NP_3_ nanoparticles with hydrodynamic radii of ~50–150 nm (Supplementary Fig. 3). The bidentate hydrogen bonding and π–π interactions derived from the catechol^27–29^ and phenyl^30,31^ end groups of the hydrophilic PEG chains provided the driving force to induce the assembly of NP_2_ and NP_3_ nanoparticles and formation of the liquid NPA_2_ and NPA_3_ coacervates (G′ < G″), respectively (Fig. 1d and Supplementary Fig. 4)^32–34^. In contrast, the control NP_1_ nanoparticles with no end groups failed to form coacervates via fluid–fluid phase separation, thereby confirming the importance of the physical interactions of end groups to the formation of NPA coacervates (Supplementary Fig. 5).

The NPA_2_ coacervate was chosen as the model for further investigations because hydrogen-bonding interactions provided by the functionalized catechol groups that drive the coacervation of NP_2_ nanoparticles can mediate adhesion of the NPA_2_ coacervate to wet tissue surfaces^35–38^. The coacervation behaviors of NPA_2_ can be visualized by fluid–fluid phase separation and further examined by transmission electron microscopy (TEM), Fourier-transform infrared spectroscopy (FTIR), and DLS (Fig. 1e). The dark spots marked by red circles in the TEM images are supposed to be the hydrophobic cores of the assembled NP_2_ nanoparticles, while the catechol-functionalized hydrophilic PEG shell acts as a flexible polymer matrix that connects the hydrophobic cores to form the stable NPA_2_ coacervate dense phase. It is noted that the free hydroxyl (-OH) groups generally exhibit narrow and intense absorption bands, while -OH groups involved in hydrogen bonding show a broader absorption band in FTIR^39,40^. The FTIR result of freeze-dried NPA_2_ coacervate showed an obvious broad -OH band, indicating the existence of hydrogen bonding interactions derived from catechol groups (Supplementary Fig. 6). DLS analysis further showed hydrogen bonding-mediated assembly of NP_2_ nanoparticles to form coacervate micro-droplets as confirmed by the increasing hydrodynamic radius of NP_2_ nanoparticles after 3.5 h of dialysis (Supplementary Fig. 7).

Free from electrostatic interactions, our noncomplex NPA_2_ coacervate is believed to be pH- and salt-independent. Compared with conventional complex coacervates that depend on pH values^21^, our NPA_2_ coacervate remained stable after 2 days and did not become a single-phase solution under a wide range of pH conditions (Fig. 1f). The frequency-dependent storage (G′) and loss (G″) moduli of the liquid NPA_2_ coacervate as revealed by rheological analysis demonstrates its highly dynamic polymeric structure due to the presence of supramolecular interactions, such as hydrogen bonding derived from the catechol groups (Supplementary Fig. 8)^41^. Furthermore, our noncomplex NPA_2_ coacervate exhibited a salting-out effect^42,43^, and the shear moduli (G′ and G″) and viscosity increased with increasing salt concentrations (Fig. 1g and Supplementary Fig. 9). It should be noted that electrostatic interactions between polyanions and polycations weaken with increasing salt concentrations; therefore, a critical salt concentration of 0.8–2.0 M NaCl generally can lead to the rapid dissociation of complex coacervates^19,22^. However, our NPA_2_ coacervate remained as a viscous liquid (G′ < G″) in 5.0 M NaCl, further confirming that the noncomplex NPA coacervation should be attributed to hydrogen bonding-induced nanoparticle assembly rather than electrostatic interactions. The rheological analysis confirmed the shear-thinning behavior of the NPA coacervates (Supplementary Figs. 8, 9). The viscosity of the NPA_2_ coacervate decreased quickly with an increasing shear rate from 0.1 to 20.0 s^−1^, thus insuring the easy injection of the NPA_2_ coacervate through a 21 G needle. In contrast, the NPA_3_ coacervate demonstrated a much lower viscosity than that of the NPA_2_ coacervate, and this can be attributed to the weak interaction energy of π–π interactions (<10 kJ mol^−1^) (Fig. 1d)^30^.

### Biocompatible and digestive enzyme-resistant NPA_2_ coacervate possesses reversible wet bioadhesion

**Fig. 2:**
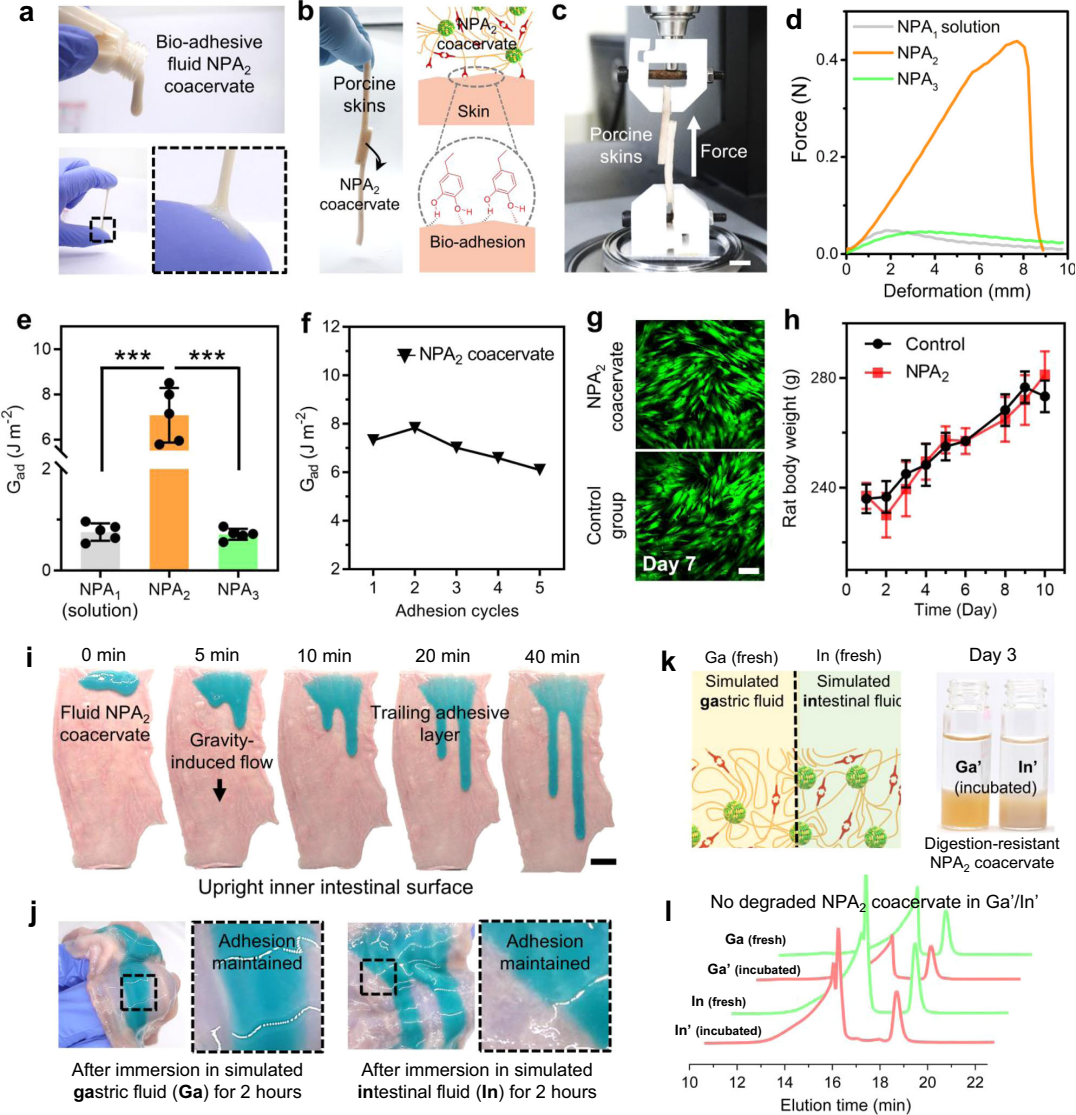
Biocompatible and fluid NPA_2_ coacervate is wet bioadhesive and resistant to digestive enzymes. **a** Fluid NPA_2_ coacervate formed a durable adhesive coating on the medical nitrile glove. **b** NPA_2_ coacervate can glue two ribbons of porcine skin tissue together and hold the weight of the tissues. **c** Schematic of the lap-shear test to measure the adhesive energy (G_ad_). Scale bar: 10 mm. **d**, **e** The adhesion performance of NPA_2_ coacervate was much better than that of the controls. NPA_2_ coacervate showed adhesive energy (G_ad_) comparable with the previously reported values (around 2–10 J m^−2^) for nanoparticle-based adhesives and polymeric adhesives. *n* = 5 independent lap-shear tests per group. **f** The hydrogen-bonding interactions derived from catechol groups ensure the stable and reversible adhesion energy level of NPA_2_ coacervate during the successive bonding/debonding cycles. **g** The viability staining of human mesenchymal stem cells (hMSCs) also suggested excellent cytocompatibility of the NPA_2_ coacervate. Scale bar: 200 μm. **h** No significant differences in body weights were observed between the rats receiving oral administration of NPA_2_ coacervate on days 1, 3, 5, 7, and 9, and untreated healthy rats during the 10-day toxicity evaluation. *n* = 3 biologically independent rats in control group, *n* = 4 biologically independent rats in NPA_2_ group. **i**, **j** The fluid NPA_2_ coacervate coating (stained by Fast Green FCF) can adhere to the fresh and wet mucosa, flow down slowly, and remain stable after soaking in simulated gastric fluid (Ga) and simulated intestinal fluid (In) at 37 °C for 2 h, respectively. Scale bar: 15 mm. **k**, **l** GPC data of the fresh (Ga, In) and used simulated gastric or intestinal fluid (Ga′, In′) incubated with NPA_2_ coacervate. No degraded or dissolved NPA_2_ coacervate components were found in the simulated gastric or intestinal fluid, respectively. Data were presented as mean ± SD. ***p < 0.001 (Ordinary one-way ANOVA). Source data are provided as a Source Data file for Fig. 2d–f, h.

Fluid NPA_2_ coacervate with a solid content of about 15 wt% flowed out from a tilted glass bottle and formed a durable adhesive coating on the medical nitrile glove (Fig. 2a and Supplementary Fig. 10). Furthermore, the catechol-mediated wet bioadhesion of NPA_2_ coacervate was sufficiently strong to glue two ribbons of the porcine skin tissue together and hold the tissue weight (Fig. 2b)^15^. The adhesive energy (G_ad_) of NPA_2_ coacervate was estimated to be ~7.07 J m^−2^, similar to the previously reported values (around 2–10 J m^−2^) for nanoparticle-based^44^ and polymeric adhesives (Fig. 2c, d, e and Supplementary Fig. 11)^45,46^. The NPA_2_ coacervate-bonded porcine skin joints can withstand an applied force five times greater than that of the homolog control groups (NPA_1_ nanoparticle solution and NPA_3_ coacervate). Furthermore, the peak adhesion force of the NPA_2_ coacervate was achieved at a much larger deformation, indicating the capability to effectively dissipate a large amount of energy under a mechanical challenge (Fig. 2d). The low adhesion energy of the control homologs can be attributed to the weak bulk cohesion (NPA_1_ solution) or interfacial adhesion (NPA_3_ coacervate), respectively. Therefore, although the π–π interaction can facilitate coacervation, the negligible bioadhesion of the NPA_3_ coacervate makes it less suitable for drug delivery in the intestinal tract. Furthermore, reversible hydrogen bonding interactions derived from the catechol groups ensure the stable adhesion energy level of the NPA_2_ coacervate during the bonding/debonding cycles (Fig. 2f). Consistent with the observed bioadhesion on porcine skins, the NPA_2_ coacervate showed decent mucoadhesion (~2.74 J m^−2^) on the luminal surface of porcine intestines (Supplementary Fig. 12), which can be attributed to the physical interactions between the catechol/PEG structure of coacervate and glycosylated mucins^47,48^.

To evaluate the safety of oral administration, we first tested the cytotoxicity of our NPA_2_ coacervate by incubating it with human mesenchymal stem cells (hMSCs). Viability staining and cell metabolic assays (MTT) of hMSCs incubated with NPA_2_ coacervate for 7 days suggested excellent cytocompatibility of the NPA_2_ coacervates (Fig. 2g and Supplementary Fig. 13). Furthermore, Sprague Dawley (SD) rats were gavaged with NPA_2_ coacervate (4 g per kg rat) every other day for 10 days. During the 10-day exposure period, the rats receiving oral administration of coacervate did not develop any abnormal symptoms such as diarrhea and showed no significant weight change compared with the untreated healthy rats, further confirming the minimal toxicity of NPA coacervates (Fig. 2h).

We next investigated the influence of the physical peristalsis and chemical environment (gastric acid and intestinal fluid) of the GI tract on NPA_2_ coacervate coating by using simulated ex vivo experiments. When deposited on the upright intestinal mucosa surface, the fluid NPA_2_ coacervate can adhere to the fresh and wet mucosa and steadily flow down driven by gravity, leaving a trailing adhesive coating layer (Fig. 2i). After soaking the NPA_2_ coacervate-coated intestinal mucosa tissues in simulated gastric fluid (Ga) or simulated intestinal fluid (In) at 37 °C for 2 h, respectively, the adherent coacervate coatings remained undiluted and maintained adhesion on the mucosa surface (Fig. 2j and Supplementary Fig. 14). The NPA_2_ coacervate also remained largely stable in the 25 mg/ml pig bile salt or under the high shear strain (up to 1000%) despite a slight dip in shear moduli toward the high shear range (Supplementary Figs. 15, 16). In addition, the digestive enzyme-resistant polymeric structure of the NPA_2_ coacervate was further confirmed by incubating NPA_2_ coacervate in simulated gastric fluid (Ga) and simulated intestinal fluid (In) at 37 °C for 3 days, respectively (Fig. 2k). GPC analysis showed that no degraded or dissolved NPA_2_ coacervate components were found in the simulated body fluids (Ga′, In’) after 3 days of incubation (Fig. 2l). It is noted that the nontoxic, biologically, and chemically inert polyurethanes are widely used in clinical practices^49,50^, and a polyurethane-based surgical adhesive (TissuGlu^®^) has been approved by United States Food and Drug Administration (FDA) for various clinical applications. Therefore, we believe that the stable polyurethane network of NPA_2_ coacervate helps establish the feasibility for the following in vivo evaluations. Besides, our laboratory-scale synthesis yielded ~300 ml of NPA_2_ coacervates per batch and showed stable batch-to-batch reproducibility, indicating the promising potential for large-scale production of NPA_2_ coacervates.

### NPA_2_ coacervate optimizes drug release with extended GI residence time in vivo

**Fig. 3:**
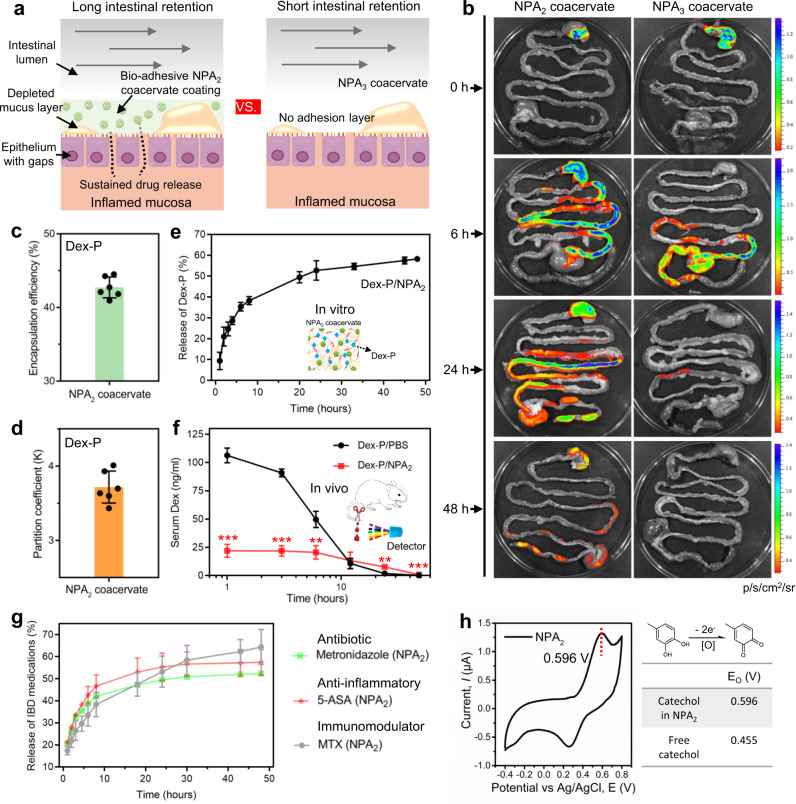
NPA_2_ coacervate demonstrates prolonged retention and mediates sustained drug release in the GI tract. **a**, **b** The bioadhesive NPA_2_ coacervate delivered via oral gavage adhered to the GI tract of SD rats for at least 2 days, whereas the control nonadhesive NPA_3_ coacervate showed short retention in the GI tract. **c**, **d** NPA_2_ coacervate has a high Dex-P encapsulation efficiency because of the hydrophobic component of its structure. *n* = 6 independent encapsulation tests. **e** NPA_2_ coacervate mediated sustained release of preloaded Dex-P over several days in vitro. **f** Dex-P-laden NPA_2_ coacervate delivered by oral gavage better sustained the serum Dex concentration at the lower therapeutic level compared with that of the oral gavage of Dex-P aqueous solution (Dex-P/PBS) in vivo. *n* = 3 biologically independent rats per group. **g**, **h** The condensed hydrophobic environment of NPA_2_ coacervate as revealed by an increased catechol oxidation potential (E_0_) of 0.596 V, facilitated the sustained release of a wide array of water-soluble small-molecular drugs including antibiotic metronidazole, anti-inflammatory 5-aminosalicylic acid (5-ASA), and immunoregulatory methotrexate disodium salt (MTX). Data were presented as mean ± SD. *p < 0.05, **p < 0.01, ***p < 0.001 (two-tailed Student’s *t*-test). Source data are provided as a Source Data file for Fig. 3c–h.

We believe that the water‐immiscible, bioadhesive, and liquid nature of our noncomplex NPA_2_ coacervate can adapt to the complex motility, fluid content, and sharp pH variation within the GI tract, and establish a large-area durable coating on the intestinal mucosa to ensure sufficient intestinal retention for sustained drug release. To further test this hypothesis, the capability of NPA coacervates as bioadhesive coatings on the gastrointestinal mucosa was further evaluated in vivo (Fig. 3a). Sprague Dawley (SD) rats with unrestricted access to water and standard laboratory diet received a single oral gavage of 1.0 ml NPA_2_ or NPA_3_ coacervate (modified with a Cy7 tag, a near-infrared fluorescent dye) and were sacrificed after 0, 6, 24, or 48 h to evaluate the fluorescence retention in the harvested GI tract (Fig. 3b). In agreement with the in vitro bioadhesive property, the NPA_2_ coacervate adhered to the rat GI tract for at least 48 h as evidenced by the intense fluorescence signal, whereas the NPA_3_ coacervate had limited retention in the GI tract after 1 day (Fig. 3b). Normal gastric emptying takes place within 2 h, and colonic arrival occurs after 5 h^51^. Therefore, the in vivo intestinal retention experiments demonstrated the prolonged retention of our NPA_2_ coacervate in the GI tract.

We further evaluated the efficacy of the NPA_2_ coacervate to mediate sustained drug release both in vivo and in vitro. We utilized Dex-P, a water-soluble sodium phosphate salt of dexamethasone (Dex), as a model anti-inflammatory drug^52^. The NPA_2_ coacervate showed a high Dex-P encapsulation efficiency (the amount of Dex-P loaded in the NPA_2_ coacervate divided by the total Dex-P input) of ~42% (Fig. 3c and Supplementary Fig. 17). The partition coefficient (K), which was defined as the ratio between the concentration of Dex-P in the NPA_2_ coacervate and the supernatant Dex-P concentration, was around 3.62 (Fig. 3d). In addition to the excellent drug loading capacity, our NPA_2_ coacervate showed prolonged release of preloaded Dex-P under both in vitro (Fig. 3e) and in vivo conditions (Fig. 3f). We next examined the drug release kinetics in vivo by monitoring the serum levels of Dex-P at selected time points after oral gavage of Dex-P-laden NPA_2_ coacervate or Dex-P aqueous solution (Fig. 3f). The serum concentration of Dex in SD rats spiked at 1 h after oral gavage of the Dex-P solution and then decreased rapidly (Dex-P/PBS). In contrast, the serum Dex concentration in the SD rats receiving oral gavage of Dex-P-laden NPA_2_ coacervate (Dex-P/NPA_2_) remained consistently at the lower therapeutic level for over 40 h (Fig. 3f). The total dosage of Dex-P administered in the two groups was the same. Therefore, our NPA_2_ coacervate can mediate sustained release and improve the pharmacokinetics of the preloaded drug in the SD rat model, thereby reducing dosing frequency and systemic drug exposure.

It is noted that ulcerative colitis is a chronic inflammatory disorder limited to the colonic and rectal mucosa^53^ and shows similar gastric and small intestinal transit time as that of healthy controls^54,55^. Because absorption of the Dex-P, a water-soluble small molecule drug, mainly occurs in the small intestine in the absence of a drug carrier^56^, the serum Dex concentrations of DSS-induced colitic rats and healthy rats receiving the equivalent amount of Dex-P in PBS (Dex-P/PBS) were similar (Supplementary Fig. 18). We further tested the capability of NPA_2_ coacervates as bioadhesive coatings to mediate the sustained release of drugs in a DSS-induced colitic rat model. The observed retention of fluorescently-label coacervate in the harvested GI tract revealed that the NPA_2_ coacervate can adhere to the GI tract of colitic rats for at least 48 h, thus confirming the similar retention time of our NPA_2_ coacervate in the GI tract of colitic rats compared with that of the healthy rats (Supplementary Fig. 19), and this explained the observed similar release and uptake kinetics of coacervate-delivered Dex-P (as indicated by serum Dex concentration) between the colitic and healthy rats (Supplementary Figs. 19, 20).

To demonstrate the versatility of NPA_2_ coacervate to mediate sustained drug release, other first-line small molecule drugs used to treat IBD including antibiotic metronidazole, anti-inflammatory 5-aminosalicylic acid (5-ASA), and immunoregulatory methotrexate disodium salt (MTX) were also encapsulated into the NPA_2_ coacervate to investigate the release kinetics (Fig. 3g). The NPA_2_ coacervate showed high encapsulation efficiency and prolonged release kinetics of these drugs (Supplementary Fig. 17). In addition, the dried drug-laden NPA_2_ coacervate that is desirable for oral administration could be prepared via lyophilization, and simply adding dried drug-laden NPA_2_ coacervate into simulated gastric fluid or water could realize the rehydration to form fluid NPA_2_ coacervate with sustained-release kinetics of diverse drugs similar to the freshly prepared drug-laden NPA_2_ coacervate before lyophilization (Supplementary Fig. 21).

We hypothesized that the capability of NPA_2_ coacervates to mediate the sustained release of water-soluble small molecules such as Dex-P can be ascribed to the relatively hydrophobic environment within the NPA_2_ coacervate. We confirmed the above hypothesis by examining the cyclic voltammetry (CV) curve of the NPA_2_ coacervate (Fig. 3h). Our NPA_2_ coacervate showed a catechol oxidation potential (E_0_) of 0.596 V in deionized water, which was significantly higher than the E_0_ of soluble small molecule 4-methylcatechol (0.455 V)^57^. The high catechol oxidation potential suggests that the NPA_2_ coacervate with a high mass ratio of hydrophobic cores provides a highly stabilizing hydrophobic environment against catechol oxidation by shielding from the aqueous solvent^58^, thus facilitating the efficient loading and sustained release of a wide array of drugs.

### Dex-P-laden NPA_2_ coacervate shows potent therapeutic efficacy against acute colitis in a rodent model

**Fig. 4:**
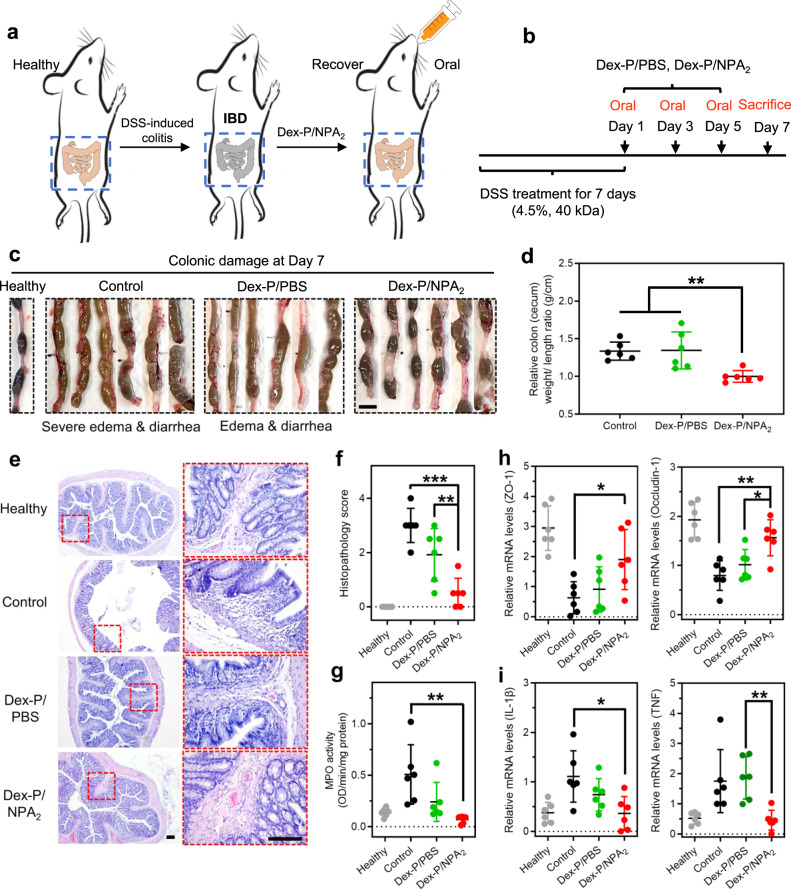
Dex-P-laden NPA_2_ coacervate demonstrates enhanced therapeutic efficacy in a rat model of DSS-induced colitis. **a**, **b** SD rats were given 4.5% DSS in drinking water to induce acute colitis. The colitic rats received oral gavages of Dex-P-laden NPA_2_ coacervates (Dex-P/NPA_2_) or the equivalent amount of Dex-P in PBS (Dex-P/PBS) on days 1, 3, and 5. Untreated colitic SD rats were used as the negative control (Control). All SD rats were sacrificed on day 7. **c**, **d** Colonic edema and diarrhea caused by DSS-induced colitis in SD rats receiving Dex-P/NPA_2_ were significantly relieved compared with that of the untreated colitic SD rats (Control) and colitic SD rats receiving Dex-P in PBS (Dex-P/PBS). *n* = 6 biologically independent rats per group. Scale bar: 10 mm. **e** Representative images of H&E staining demonstrated that histological inflammation was diminished in the colitic SD rats receiving Dex-P/NPA_2_, while histological damages were observed in untreated colitic SD rats (Control) or colitic SD rats receiving Dex-P/PBS. Scale bar: 150 μm. **f**–**i** On day 7, colon tissues were analyzed for histopathology score **f**, MPO activity **g**, mRNA levels of tight junction-associated proteins including ZO-1 and occludin-1 **h**, and pro-inflammatory cytokines including interleukin *IL*-*1β* and TNF **i**. Data were presented as mean ± SD. *n* = 6 biologically independent rats per group. *p < 0.05, **p < 0.01, ***p < 0.001 (Ordinary one-way ANOVA). Source data are provided as a Source Data file for Fig. 4d, f–i.

We next evaluated the therapeutic efficacy of the Dex-P-laden NPA_2_ coacervate in the rat model of dextran sulfate sodium (DSS)-induced colitis (Fig. 4a). SD rats weighing around 250 g were given 4.5% DSS in drinking water for 7 days to develop acute colitis^25^. Clinical manifestations of the colitis, such as severe rectal bleeding, watery diarrhea, and colonic edema were observed after 7 days (Supplementary Fig. 22). After successfully establishing the colitis model in rats, colitic SD rats received oral gavages of Dex-P-laden NPA_2_ coacervates (Dex-P/NPA_2_) or the equivalent amount of Dex-P in PBS (Dex-P/PBS) on days 1, 3, and 5 (Fig. 4b). Untreated colitic SD rats were used as the negative control. All SD rats were allowed unrestricted access to water and standard laboratory diet before and after oral gavage and sacrificed on day 7 for further evaluation of colon weight and length, histological severity, IBD-associated colonic myeloperoxidase (MPO)-activity, mRNA levels, and protein expressions of tight junction-associated proteins (ZO-1 and occludin-1) and pro-inflammatory cytokines, such as interleukin *IL*-*1β* and tumor necrosis factor (TNF) in the distal colon.

Our results demonstrated the significant therapeutic efficacy of Dex-P/NPA_2_ treatment against DSS-induced acute colitis. Dex-P-laden NPA_2_ coacervate significantly alleviated colonic edema and diarrhea caused by DSS-induced acute colitis (Fig. 4c). Relieved edema in colitic SD rats receiving Dex-P/NPA_2_ was further confirmed by the lower colon (cecum) weight/length ratio (Fig. 4d). Representative images of hematoxylin and eosin (H&E) staining demonstrated significantly reduced histological inflammation in the colitic SD rats receiving Dex-P/NPA_2_, while histological damages, such as the compromised integrity of the mucosal epithelial lining, decrease in villus height and crypt depth, interstitial edema, and inflammatory infiltration were observed in untreated colitic SD rats (Control) or rats treated with the equivalent amount of Dex-P solution in PBS (Dex-P/PBS, Fig. 4e). Furthermore, histopathology scoring of H&E-stained tissue sections was used to evaluate the severity of colonic histological damage in a blinded fashion by a trained pathologist. Disease severity in colitic SD rats receiving Dex-P/NPA_2_ decreased significantly (mean histopathology score, 0.500) compared with colitic SD rats in the Dex-P/PBS group (mean histopathology score, 1.917) and the untreated control group (mean histopathology score, 3.000). Histopathology scores of colitic SD rats in the Dex-P/PBS group were not significantly different from the untreated control group (*P* = 0.056, Fig. 4f).

Colonic MPO activity in colitic SD rats receiving Dex-P/NPA_2_ was also significantly reduced compared with the untreated control group (Fig. 4g)^59^. Although we also observed a reduction of MPO activity in the Dex-P/PBS group due to the therapeutic activity of Dex-P against IBD, the high serum Dex level associated with such administration of Dex-P aqueous solution indicates an increased risk of complications related to severe systemic drug exposure (Fig. 3f). We have also tested the therapeutic efficacy of NPA_2_ alone to treat colitic rats via oral gavage on days 1, 3, and 5 and then analyzed the MPO activity and histopathology score of colon tissues on day 7 (Supplementary Fig. 23). The therapeutic efficacy of treatment with NPA_2_ alone did not show a significant difference compared with that of the non-treated colitic rats (Control) as evidenced by the similar MPO activity, histopathology score, and H&E staining, thus confirming that the NPA_2_ coacervate only served as a drug delivery vehicle and did not mitigate the colitic symptoms. In addition, colitic SD rats receiving Dex-P/NPA_2_ recovered the expression of tight junction-associated proteins including ZO-1 and occludin-1 and showed significantly reduced local levels of IL-1β, IL-6, and TNF compared with the untreated group and Dex-P/PBS group (Fig. 4h, i and Supplementary Figs. 24,25). Taken together, oral delivery of Dex-P encapsulated in NPA_2_ coacervate to colitic SD rats showed significantly enhanced therapeutic outcomes and reduced systemic exposure than administering the equivalent amount of Dex-P in an aqueous solution (Supplementary Fig. 26).

### Dex-P/NPA_2_ regulates innate immune responses and restores the gut microbiota

**Fig. 5:**
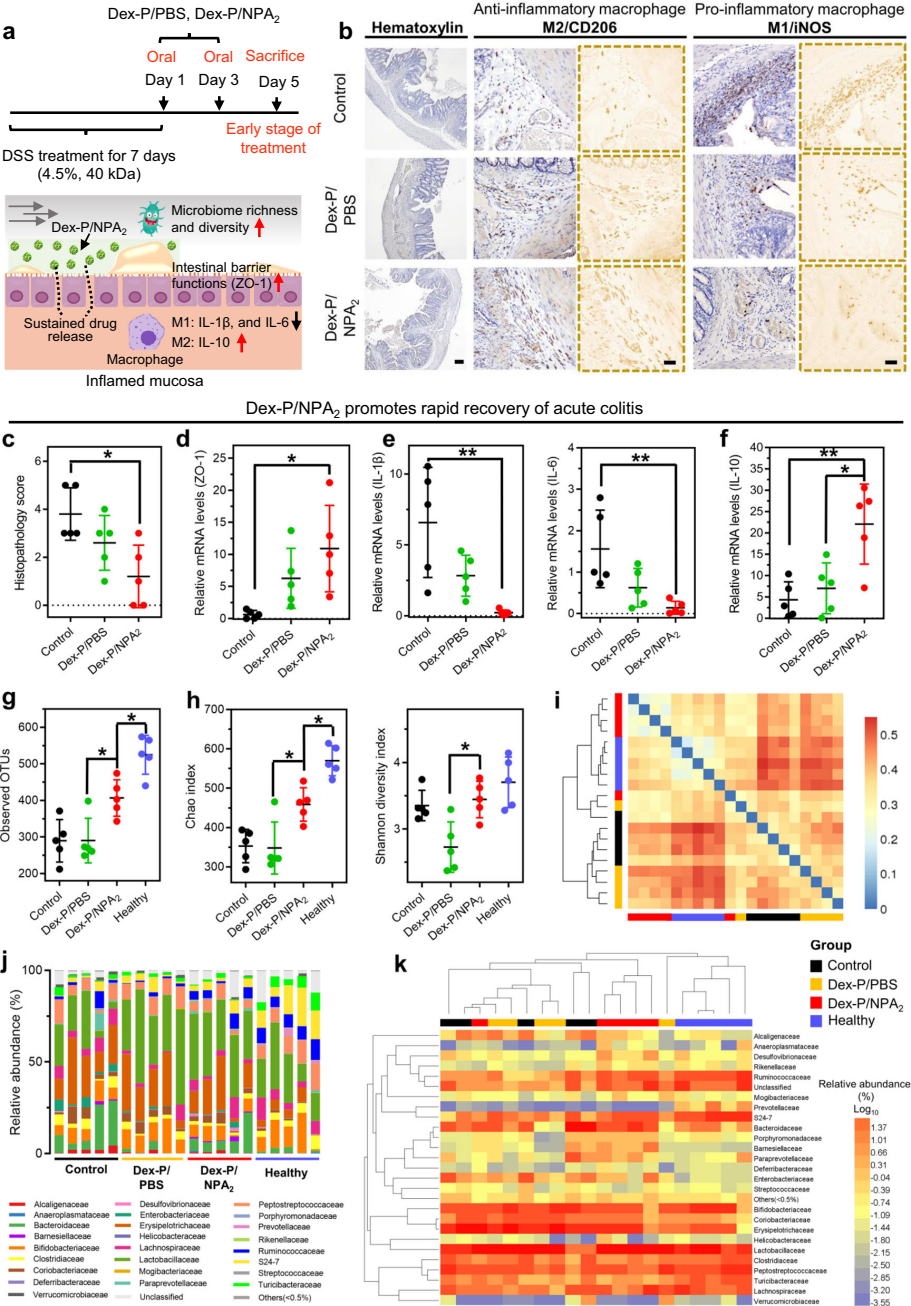
Dex-P/NPA_2_ treatment regulates the innate immune responses and restores the gut microbiota. **a**–**f** On day 5, colitic SD rats receiving Dex-P/NPA_2_, or Dex-P/PBS, and untreated colitic SD rats (Control) were sacrificed, and colon tissues were analyzed for macrophage polarization. **b** Immunohistochemical staining against key macrophage M1/M2 polarization markers iNOS/CD206, **c** histopathology score, **d** mRNA levels of tight junction-associated protein ZO-1, **e** pro-inflammatory cytokines including interleukin *IL*-*1β* and IL-6 released by M1 macrophages, and **f** anti-inflammatory cytokine IL-10 released by M2 macrophages. Scale bar for hematoxylin staining, 200 μm. Scale bar for immunohistochemistry staining, 50 μm. *n* = 5 biologically independent rats per group. **g**–**k** Fecal samples collected on day 5 from colitic SD rats were analyzed for gut microbiota by sequencing the V4 region of the 16 S rRNA gene. Dex-P/NPA_2_ treatment increased **g** bacterial richness (observed operational taxonomic units, OTUs), **h** Chao diversity, and **h** Shannon diversity in colitic SD rats compared with colitic SD rats in the Dex-P/PBS group and the untreated colitic rats (Control). *n* = 5 biologically independent rats per group. **i** A clustered heatmap of UniFrac values for measuring gut microbiota β-diversity illustrated that colitic SD rats receiving Dex-P/NPA_2_ and healthy SD rats were clustered more closely, suggesting more similar bacterial compositions. The color of the square shows the distance of evolution between each two samples. The range of blue to red corresponds to close to far distance, and the bigger index means the greater differences between samples. **j** Taxonomic bacterial distribution histogram and **k** clustered heatmap based on the relative abundance (Log_10_) of the gut microbiota at the family level are presented. The upper longitudinal clustering indicates the similarity of gut microbiota among individual SD rats. The closer distance and shorter branch length indicate more similar gut microbiota between the SD rats. Data were presented as mean ± SD. *p < 0.05, **p < 0.01, ***p < 0.001 (Ordinary one-way ANOVA). Source data are provided as a Source Data file for Fig. 5c–k.

Macrophage activation can be broadly classified into the pro-inflammatory M1 polarization or anti-inflammatory M2 polarization^60^, and macrophages play a key role in the maintenance of mucosal homeostasis by secreting many cytokines^61^. Therefore, we next investigated the intestinal immune responses of colitic SD rats by analyzing macrophage polarizations in the harvested colon tissues from colitic rats receiving Dex-P/NPA_2_ or Dex-P/PBS and untreated colitic SD rats (Control) on day 5 (Fig. 5a). Many studies have shown that dexamethasone (Dex), a corticosteroid, can promote anti-inflammatory M2 macrophage polarization and suppress pro-inflammatory M1 polarization^62–64^, thus enhancing the secretion of anti-inflammatory cytokines, such as IL-10^65^. Immunohistochemistry staining against CD206 (M2 marker) or iNOS (M1 marker) showed increasing M2 macrophage polarization in the colitic SD rats receiving Dex-P/NPA_2_ (Fig. 5b and Supplementary Fig. 27). Furthermore, Dex-P/NPA_2_ treatment significantly reduced the local levels of pro-inflammatory IL-1β and IL-6 released by M1 macrophages (Fig. 5e) and increased the level of anti-inflammatory IL-10 (Fig. 5f)^66^. Taken together, oral administration of Dex-P/NPA_2_ to colitic SD rats promoted the M2 polarization of intestinal macrophages^61^ and mediated strong anti-inflammatory effects against acute colitis, thereby creating a favorable immune microenvironment to promote colon repair and regeneration (Fig. 5c, d).

Dysbiosis of gut microbiota, which is implicated in various inflammatory and immune diseases^67^, plays a key role in the pathogenesis of IBD, while the chronic inflammation caused by IBD, in turn, promotes dysbiosis of gut microbiota^68^. Therefore, restoring gut microbiota and recovery of gut immune homeostasis can complement each other in the successful treatment of acute colitis. Although orally administered Dex-P may not directly modulate the gut microbiota^69^, we expected the enhanced gut immune microenvironment by Dex-P/NPA_2_ treatment can positively regulate the gut microbiota. Analyses of fecal samples collected from colitic SD rats on day 5 by sequencing the V4 region of the 16 S ribosomal ribonucleic acid (rRNA) gene showed that Dex-P/NPA_2_ treatment indeed increased the bacterial richness (observed operational taxonomic units, OTUs) and diversity (Chao and Shannon indices) in colitic SD rats (Fig. 5g, h). In addition, a heatmap of β-diversity distance distribution was generated, and samples with similar β-diversity were clustered to reflect the similar compositions of gut microbiota (Fig. 5i). The β-diversities between colitic SD rats receiving Dex-P/NPA_2_ and healthy SD rats were clustered more closely compared with colitic SD rats in the Dex-P/PBS group and the untreated control group (Control), thus suggesting the enhanced recovery of gut microbiota in colitic SD rats by Dex-P/NPA_2_ treatment. This was further confirmed by the taxonomic bacterial distribution histogram and clustered heatmap based on the relative abundance of gut microbiota at the family level (Fig. 5j, k).

In conclusion, we have developed a simple yet highly efficient strategy for oral drug delivery by using a nanoparticle-assembled (NPA) bioadhesive noncomplex coacervate. The NPA coacervate exhibited: (1) pH and salt-independent stability under the complex environment within the GI tract; (2) effective coating of large intestinal surface and prolonged intestinal retention for several days; and (3) enhanced therapeutic efficacy for IBD and gut microbiota restoration with reduced systemic drug exposure via the controlled release of a loaded drug. When used for clinical treatments, these highly desired attributes of NPA coacervate-based oral drug delivery will potentially enhance patient compliance and acceptance and therefore the eventual therapeutic outcomes, especially compared with conventional therapies such as enema, subcutaneous and intravenous injection. Although we used Dex-P as a model drug to treat IBD in this study, our bioadhesive NPA_2_ coacervate can effectively deliver many other drugs, especially the water-soluble small molecule drugs, which are usually difficult to achieve sustained-release kinetics by using the existing oral drug delivery platforms. Therefore, we believe that the NPA coacervate-based drug delivery represents a promising approach to the treatment of numerous gastrointestinal diseases, including mucosal wound healing, gastrointestinal cancers, peptic ulcers, and viral infections.

## Methods

### In situ assembly of as-prepared core-shell nanoparticles into NPA coacervate

The concentrated amphiphilic polymers composed of hydrophilic poly(ethylene glycol) (PEG) and hydrophobic segments in 20 ml DMF (~27 wt%) were dropped into cold deionized water (60 mL) with 600 rpm magnetic stirring to form the core-shell nanoparticles (NP_1_, NP_2_, and NP_3_), respectively. The nanoparticle solutions then were dialyzed against deionized water (standard regenerated cellulose (RC) dialysis membrane with 3.5 kDa cutoff, Spectrum Chemical) at room temperature for 24 h to form NPA coacervates via nanoparticle assembly. To determine the successful synthesis and the molecular weight of the hydrophobic segment of the amphiphilic polymers, 1.5 μL of the hydrophobic segment in methanol (1 mg/ml) was premixed with 1.5 μL of matrix solution (saturated α-cyano-4-hydroxycinnamic acid, Sigma). Then this mixture was applied to a ground steel target that was ready for mass spectrometry. Finally, the analyses of air-dried samples were performed in an AutoFlex Speed LRF MALDI-TOF mass spectrometer (Bruker Daltonics, Germany) under reflector mode.

### In vitro biocompatibility test

Passage 4 hMSCs (8 × 10^4^ cells/well, Lonza, Catalog #: PT-2501) were incubated in six-well plates. Each well was added 2 mL growth medium consisting of α-minimum essential medium (Gibco) supplemented with 16.7% fetal bovine serum (FBS, Gibco), 1% glutamine (Sigma), and 1% penicillin/streptomycin (Sigma). NPA_2_ coacervates with a final concentration of 5 mg/mL were added. The control groups were not added NPA_2_ coacervates. The medium was changed every 2 days. Live/dead staining (Thermo Fisher) was performed on day 7. hMSCs were imaged using a confocal laser scanning microscope (Nikon C2). For the MTT assay, hMSCs (0.6 × 10^4^ cells/well) were incubated in a 96-well plate in the growth medium. About 5 mg/mL NPA_2_ coacervates were used to culture the cells for 7 days. After that, 10 μL MTT (0.5 mg/mL) was added to each well to produce formazan crystals according to the manufacturer’s instructions (Thermo Fisher), which were further dissolved by 200 μL of DMSO (Sigma). Finally, a Multiskan microplate reader (Thermo Fisher) was used to collect the absorbance value of each well at 540 nm for cell metabolic calculation.

### Animals

Sprague Dawley (SD) rats (200–300 g, female, 8–12 weeks) were obtained from the Laboratory Animal Services Centre (Chinese University of Hong Kong) and cohoused for a week before being randomly assigned to different groups. Animal experiments were approved by the Animal Experimentation Ethics Committee of the Chinese University of Hong Kong (19-152-MIS) and were carried out in accordance with the guidelines of the Animals Ordinance (Chapter 340), Department of Health, Hong Kong.

### Oral toxicity test

SD rats (200–300 g, female, 8–12 weeks) receiving oral administration of NPA_2_ coacervate (4 g per kg rat) on days 1, 3, 5, 7, and 9 during the 10-day toxicity evaluation and untreated healthy rats were used as the control group. Body weights were measured. Any abnormal symptoms such as diarrhea were recorded.

### Ex vivo adhesion experiments

Fresh intestinal tissues were bought from Sha Tin Market (Hong Kong), stored on ice, and washed with PBS buffer (1×) three times before use. For ex vivo adhesion testing, NPA_2_ coacervate stained by Fast Green FCF (Sigma) was deposited on the upright wet intestinal mucosa surface to monitor gravity-induced fluid adhesion behaviors of NPA_2_ coacervate over time. Then the above NPA_2_ coacervate-coated intestinal tissues were soaked in simulated gastric fluid (Ga) or simulated intestinal fluid (In) at 37 °C for 2 h respectively. To monitor changes of the adherent coacervate coatings, photos were taken before and after the soaking and analyzed by ImageJ 1.8.0_172. Simulated gastric fluid (Ga) and simulated intestinal fluid (In) with enzymes were prepared following United States Pharmacopoeias (USP).

### Digestive enzyme-resistant test

NPA_2_ coacervates were incubated in simulated gastric fluid (Ga) and simulated intestinal fluid (In) at 37 °C for 3 days, respectively. GPC (Agilent system, 1260 Infinity II) with a refractive index detector was used to detect whether the degraded or dissolved NPA_2_ coacervate components existed in the simulated body fluids (Ga′, In′) after 3 days of incubation. PBS (1×, Gibco) was used as the elution phase. NPA_2_ coacervate was also placed in 1x PBS buffer with pig bile salt (25 mg/ml, Solarbio) for 24 h at 37 °C to test its stability.

### In vivo NPA coacervates adhesion experiments

Sprague Dawley (SD) rats (female, 8–12 weeks) weighing around 250 g were housed in groups of three rats per cage. SD rats were allowed unrestricted access to water and a standard laboratory diet before and after oral gavage. SD rats weighing around 250 g received 4.5% Dextran sodium sulfate (DSS, 40 kDa, Alfa Aesar) supplemented in the drinking water for 7 days to induce colitis, followed by normal water during the treatment. Healthy SD rats or DSS-induced colitic rats received a single oral gavage of 1.0 ml NPA_2_ or NPA_3_ coacervate (modified with a Cy7 tag, a near-infrared fluorescent dye). Briefly, an amine-NCO reaction between Cyanine 7 amine (0.2 mg, Lumiprobe) and polymerized PEG with terminated NCO groups (1 at Supplementary Method 1) was used to modify the hydrophilic PEG shell of NP nanoparticles with a Cy7 tag. Then SD rats were sacrificed after 0, 6, 24, or 48 h, and GI tracts were harvested to evaluate the fluorescence retention by an in vivo live imaging system IVIS200 (Xenogen) with an ICG filter channel to measure the fluorescent signal intensity.

### In vitro and in vivo drug release

For in vitro Dex-P release, 2 ml of Dex-P in PBS (1.5 mg/ml, TCI) was added to 1 ml NPA_2_ coacervate followed by vortex for 15 s for Dex-P encapsulation. The changes of supernatant Dex-P concentrations were measured by a Shimadzu UV-3600 UV–vis–NIR spectrophotometer at 242 nm. Then 2 ml fresh PBS buffer (1×, Gibco) was added to the as-prepared Dex-P-laden NPA_2_ coacervate (1 ml). Dex-P concentrations in the above PBS were measured at 242 nm. The protocols for in vitro release of other first-line small molecule drugs used to treat IBD including antibiotic metronidazole (Metro, J&K), anti-inflammatory 5-aminosalicylic acid (5-ASA, J&K), and immunoregulatory methotrexate disodium salt (MTX, Alfa Aesar) were the same as in vitro Dex-P release, but the release of Metro, 5-ASA, and MTX was measured at 320, 331, and 371 nm, respectively.

For in vivo Dex-P release, healthy SD rats or DSS-induced colitic rats (200–300 g, female, 8–12 week) received a single oral gavage of 1.0 ml as-prepared Dex-P-laden NPA_2_ coacervate (Dex-P/NPA_2_) or Dex-P aqueous solution (Dex-P/PBS). The total dosage of Dex-P administered in the two groups was the same (~1.15 mg). SD rats were allowed unrestricted access to water and a standard laboratory diet before and after oral gavage. Blood was collected from the tail vein at selected time points after oral gavage of Dex-P-laden NPA_2_ coacervate or Dex-P aqueous solution to measure the serum Dex concentrations by a commercial Dexamethasone ELISA assay (Neogen Corporation) following the manufacturer’s instructions.

### DSS-induced rat models of colitis

SD rats (female, 8–12 weeks) weighing around 250 g received 4.5% Dextran sodium sulfate (DSS, 40 kDa, Alfa Aesar) supplemented in the drinking water for 7 days to induce colitis, followed by normal water during the treatment^25^. Healthy SD rats were provided with normal water only. Then NPA_2_ coacervate only, Dex-P-laden NPA_2_ coacervates (Dex-P/NPA_2_) or the equivalent amount of Dex-P (1.15 mg, MCE, USA) in PBS (Dex-P/PBS) on days 1, 3, and 5 was administered via an oral route into colitic SD rats. We have also tried to treat DSS-induced colitic rats with the equivalent amount of Dex-P (1.15 mg, MCE, USA) in PBS (Dex-P/PBS) via enema on days 1, 3, and 5. Dex-P was used at 1.15 mg per dose based on the published reports related to treatments in rodent colitis models^52,70,71^. Untreated colitic SD rats were used as a negative control. All SD rats were allowed unrestricted access to water and a standard laboratory diet before and after oral gavage. All SD rats were sacrificed on day 7, and the entire colon was excised and gently washed with physiological saline for further evaluation of colon weight and length, histological severity, IBD-associated colonic myeloperoxidase (MPO)-activity, expression of related mRNAs, immunofluorescence staining against tight junction-associated proteins including ZO-1 and occludin-1 (Santa Cruz), and ELISA tests for IL-6 and TNF (MultiSciences).

On day 5 of the treatment, colon tissues from colitic rats receiving Dex-P/NPA_2_ or Dex-P/PBS on days 1, and 3 and untreated colitic SD rats (Control) were harvested to study the intestinal immune responses of colitic SD rats by analyzing macrophage polarization. And fresh fecal samples were collected from the above colitic SD rats in each group on day 5 and stored at −80 °C in autoclaved sterile vials for further analysis of gut microbiota.

### Immunol staining

Immunohistochemical staining for macrophage polarization was performed using an antibody against CD206 (M2 marker, Abcam, ab64693, dilution of 1:200) or iNOS (M1 marker, Abcam, ab283655, dilution of 1:200), followed by Vectastain ABC Kit (Vector Laboratories) and the DAB (3, 3-diaminobenzidine) Substrate Kit (Vector Laboratories). Briefly, paraffin sections (7-μm thick) of distal colon tissues were deparaffinized and rehydrated. Then antigen retrieval of sections was performed in microwave-assistant hot antigen retrieval buffer (1×, citrate buffer pH 6.0, Abcam). Sections were further incubated with 10% horse serum (Thermo Fisher) for 30 min to block nonspecific binding sites. Next sections were incubated at 4 °C overnight with primary antibody directed against CD206 or iNOS (dilution of 1:200), followed by incubation with a biotinylated secondary antibody (Abcam, ab207995, dilution of 1:500). The staining was further developed using the Vectastain ABC Kit (Vector Laboratories) and the DAB (3, 3-diaminobenzidine) Substrate Kit (Vector Laboratories) for peroxidase. Finally, sections were stained with hematoxylin.

Immunofluorescence staining for tight junction-associated proteins was performed using antibodies against ZO-1 (Santa Cruz, sc-33725) and occludin-1 (Santa Cruz, sc-271842), respectively. After rehydrating and performing antigen retrieval of the paraffin sections (7-μm thick) of distal colon tissues, nonspecific binding sites were blocked by 10% horse serum (Thermo Fisher) for 30 min. Next, sections were incubated at 4 °C overnight with primary antibodies directed against ZO-1 conjugated with Alexa Fluor^®^ 488 (dilution of 1:200) and occludin-1 conjugated with Alexa Fluor^®^ 594 (dilution of 1:200). Nuclei were then stained with DAPI (4′,6-diamidino-2-phenylindole) (Thermo Fisher, dilution of 1:1000). Immunofluorescence images were obtained by using a confocal laser scanning microscope (Nikon C2).

### Statistical analysis

All data were presented as mean ± standard deviation. Statistical analyses were performed by using ordinary one‐way ANOVA with Tukey’s post hoc testing or two-tailed Student’s *t*-test to compare multiple groups or two groups (Graphpad Prism 7.0), respectively. *P* values less than 0.05 were considered as statistically significant differences among the compared groups, to which different asterisks were assigned (*p < 0.05; **p < 0.01; ***p < 0.001). For all multiple comparisons, compare the mean of each column with the mean of every other column.

### Reporting Summary

Further information on research design is available in the Nature Research Reporting Summary linked to this article.

## Supplementary information

Supplementary Information

Reporting Summary

## Data Availability

The datasets for gut microbiota generated in this study have been deposited in the GEO repository under accession code “GSE167138”. Ribosomal Database Project (RDP) Classifier is “publicly available [https://sourceforge.net/projects/rdp-classifier/files/rdp-classifier/]”. All other relevant data supporting the key findings of this study are available within the article and its Supplementary Information files or from the corresponding author upon reasonable request. Source data are provided with this paper.

## Source data

Source Data
