# Supplementary material for: Nanoparticle-assembled bioadhesive coacervate coating with prolonged gastrointestinal retention for inflammatory bowel disease therapy
Source: Nat Commun. 2021 Dec 9;12:7162. doi: 10.1038/s41467-021-27463-6 (PMC8660811; doi:10.1038/s41467-021-27463-6)
Supplement: Supplementary file 1 — Supplementary Information [file 41467_2021_27463_MOESM1_ESM.pdf]

Supplementary Materials for

Nanoparticle-Assembled Bioadhesive Coacervate Coating with Prolonged Gastrointestinal Retention for Inflammatory Bowel Disease Therapy

Pengchao Zhao[1,7], Xianfeng Xia[2,3,7], Xiayi Xu[1,7], Kevin Kai Chung Leung[4], Aliza Rai[4], Yingrui Deng[1], Boguang Yang[1], Huasheng Lai[4], Xin Peng[1], Peng Shi[5,6], Honglu Zhang[5,6], Philip Wai Yan Chiu[1,3,4*], Liming Bian[5,6*]

[7]These authors contributed equally: Pengchao Zhao, Xianfeng Xia, Xiayi Xu.

email: philipchiu@surgery.cuhk.edu.hk; bianlm@scut.edu.cn

This PDF file includes:

Supplementary Methods

1. Synthesis of amphiphilic polymers of nanoparticles (NP_1, NP_2, and NP_3)
2. Characterization of nanoparticle-assembled NPA_2 coacervate
3. Rheological time sweep under various salt concentrations
4. Lap shear adhesion measurement
5. Cyclic voltammetry (CV) measurement of NPA_2 coacervate
6. Histological analysis
7. MPO activity measurement
8. Real time RT-PCR
9. Gut microbiota analysis

Supplementary Figures 1-27

Supplementary References

Supplementary Methods

1. Synthesis of amphiphilic polymers of nanoparticles (NP_1, NP_2, and NP_3)

First, anhydrous polyethylene glycol (PEG, 5g) with an average molecular weight of 1000 (Alfa Aesar) and isophorone diisocyanate (1.389 g, 6.25 mmol, IPDI, Sigma) were added to a dry three-necked, round-bottomed flask. Under nitrogen protection, the above mixture was first polymerized for 30 min at 60 °C. Then 20 μL catalyst, dibutyltin dilaurate (DBTDL, 0.034 mmol, Sigma) was added to the above system to react for another 45 min at 80 °C. After cooling the mixture to room temperature, 50 mL anhydrous THF (J&K) was then added to dissolve the above polymerized PEG (1) with terminated NCO groups. Second, dopamine hydrochloride (50 mg, 0.264 mmol, J&K) in 5 mL anhydrous DMF (J&K) was added, and anhydrous triethylamine (26.7 mg, 0.264 mmol, J&K) was subsequently added to react for 30 min to form the end group-modified hydrophilic PEG shell (2) of NP_2 nanoparticles. In contrast, β-phenylethylamine (32 mg, 0.264 mmol, Sigma) was added to the polymerized PEG with terminated NCO groups (1) to form the end group-modified hydrophilic PEG shell (3) of NP_3 nanoparticles, while unmodified polymerized PEG with terminated NCO groups (1) was directly used as the hydrophilic PEG shell of NP_1 nanoparticles. Third, 650 mg hydrophobic segment (4) than can act as the hydrophobic core of nanoparticles in 10 mL anhydrous DMF was added to react with hydrophilic PEG shell (1, 2, and 3) for another 1 h to form the final amphiphilic polymers of nanoparticles (NP_1, NP_2, and NP_3), respectively.

Hydrophobic segment (4) that can act as the hydrophobic core of nanoparticles was synthesized in a simple Ugi four-component reaction, which is a highly efficient and atom economic reaction under mild conditions for the synthesis of polymeric products[1, 2]. Under nitrogen protection, 3,4-dihydroxybenzaldehyde (7.873g, 57 mmol, J&K) in 25 mL methanol was injected into the solution of 1,6-hexanediamine (3.312 g, 28.5 mmol, TCI) in another 25 mL methanol under magnetic stirring. After 1 hour, tert-butyl isocyanide (5 g, 60 mmol, J&K) and hexanoic acid (4.165 mg, 28.5 mmol, TCI) were added to the above system. Then the reaction mixtures were stirred for 96 hours at room temperature. Precipitation in diethyl ether (1000 mL) yielded the above hydrophobic segment (5).

2. Characterization of nanoparticle-assembled NPA_2 coacervate

The molecular weight distribution of the above polymerized NCO-terminated PEG (1, 1 mg/ml) of which the residual NCO groups had been neutralized by 1 ml anhydrous methanol (J&K) for 24 h, was determined by GPC (Agilent system, 1260 Infinity II) with a refractive index detector. Dimethylformamide (DMF, GPC grade) was used as the elution phase, and polystyrene standards (Agilent, EasiVial PS-M) ranging from 945 to 364000 g/mol were used as the calibration.

Dynamic light scattering (DLS) results of as-prepared nanoparticles were obtained with a DelsaMax pro particle sizing & zeta potential instrument. The as-prepared nanoparticles in transparent solutions (DMF/ deionized water = 3/8, v/v) were diluted to a concentration of 0.5 wt% by adding a mixture solvent (DMF/ deionized water = 3/8, v/v). Then DLS was performed to detect the hydrodynamic radius of as-prepared nanoparticles. The diluted NP_2 nanoparticles (0.5 wt%) were further dialyzed against deionized water (RC dialysis membrane with 3.5 kDa cutoff, Spectrum Chemical) at room temperature for 3.5 h, and DLS was performed again to confirm the assembly of NP_2 nanoparticles.

The formation of NPA_2 coacervate via NP_2 nanoparticle assembly was further verified by transmission electron microscopy (TEM). NPA_2 coacervate was dropped on the surface of DI water, floated, and expanded into a thin liquid film. 400-mesh copper TEM grids (Electron Microscopy Sciences) were used to pick up the thin NPA_2 coacervate layer and followed by drying under room temperature. Then the prepared samples were visualized by TEM (Hitachi) operating at 100 kV. Attenuated total reflectance–Fourier transform infrared (ATR-FTIR, Cary 630, Agilent) spectroscopy was used to analyze the absorption of freeze-dried NPA_2 coacervate.

3. Rheological time sweep under various salt concentrations

The rheological time sweeps of the NPA_2 coacervates were performed on a Kinexus rheometer (Malvern) at room temperature by using a 20 mm 1° cone. For time sweep, the 1% strain, 1Hz frequency were used. NPA_2 coacervates were first immersed into NaCl (0.1, 0.5, 1, 2 and 5 M, J&K) and Na_2SO_4 (0.1, 0.5 and 1M, J&K) aqueous solutions for 1 hour, respectively. Then time sweeps were performed to evaluate the changes of G”, G’ and viscosity.

4. Lap shear adhesion measurement

Lap-shear tests were performed according to the previous report on a Kinexus rheometer (Malvern) equipped with a 20 N load cell (100 mm min^{-1}) [3]. Fresh porcine skins and porcine intestine were bought from Sha Tin Market (Hong Kong), and washed with PBS buffer (1×, Gibco) three times before use. The NPA_2 and NPA_3 coacervates or single-phase solutions of NP_1 were spread over the interface between two ribbons of skin tissue or porcine intestine luminal surfaces before lap-shear tests, respectively. Then the lap-shear tests were conducted immediately. Reversible bonding/debonding cycles of NPA_2 coacervate were performed by directly re-sticking two ribbons of porcine tissue together for the next lap-shear test without a pause. Adhesion energy, G_{ad}, was calculated using the following equation[4]:

$$G_{ad} = 3(F/w)^2/(2Eh)$$

F is the measured adhesive failure force. w and h denote, respectively, the width and thickness of the pork skin ribbon. E is the tensile modulus of pork skin (w = 5 mm, h = 3.5 mm). We have calculated the E values by performing the tensile stress-strain curve of the porcine skin (~0.53 MPa) and the porcine intestine (~0.705 MPa), respectively (Supplementary Figure 11 and 12).

5. Cyclic voltammetry (CV) measurement of NPA_2 coacervate

CV was performed on a VMP3 electrochemical testing unit (Bio-Logic)[5]. The analysis was carried out using a three-electrode cell: a Pt wire as the counter electrode, an Ag/AgCl reference electrode, and a glassy carbon electrode (GCE) served as the working electrode. Scan rate 0.05 V s^{-1} was used. Measurements were done with 0.01 wt% NPA_2 coacervates in deionized water.

6. Histological analysis

Tissues were first fixed in PBS buffer (1×) with 4% paraformaldehyde (J&K). Then tissue sections of the distal colon embedded in paraffin were stained using haemotoxylin and eosin (H&E, Sigma). Furthermore, histopathology scoring of H&E-stained tissue sections was used to evaluate the severity of colonic histological damage in a blinded fashion by a trained pathologist[6]. Briefly, DSS-induced colonic damage was scored as follows: 0, Normal; 1, slight increase in cellularity; 2, increased cellularity including neutrophils, mild edema; 3, focal erosions, ulcerations of the

mucosa; 4, large and or multifocal mucosal ulcerations; 5, Loss of mucosal architecture.

7. MPO activity measurement[7]

A colon segment (1:20, w/v) in 50 mM phosphate buffer (pH 6.0) containing 0.5% hexadecyltrimethyl ammonium bromide was homogenized on ice using plastic homogenization pestles. After sonicating for 15 s, the homogenate was centrifuged at 19721 x g (Centrifuge 5424 R, Eppendorf) for 15 min. The supernatant (10 μl) was added to 190 μl of 50 mM phosphate buffer (pH 6) containing 0.167 mg/ml O-dianisidine dihydrochloride and 0.0005% hydrogen peroxide[8]. The changes in absorbance at 460 nm (25 ℃) were measured every 5 minutes. MPO activity was expressed as changes of OD values every minutes per mg of protein. The overall protein content was measured by Pierce BCA Protein Assay Kit (Thermo Fisher Scientific).

8. Real time RT-PCR

RNA was extracted from colonic tissues using TRIzol (Thermo Fisher). The RNA concentration was then determined by the ND-100 spectrophotometer (Nanodrop Technologies). Reverse transcription was performed by PrimeScript RT Master Mix (Takara Bio). Quantitative reverse transcription polymerase chain reaction (qRT-PCR) was performed using the SYBR Green method (Takara Bio) on Applied Biosystems 7300 Real-Time PCR system. The gene expression levels were normalized to those of GAPDH by the ΔΔCT relative method. The customized qPCR Primers were bought from GenScript (China). The following primer sets were used for amplifications:

qPCR Primer		
Gene	Forward (5'→3')	Reverse (5'→3')
ZO-1	GCAAGGGATAGAAGCGCAAG	TATGGCTGGCCAATCGAAGAC
Occludin-1	TAGCCATTGTCCTGGGGTTC	CGGTCCATCTTTCTTCGGGT
IL-1β	CAGGATGAGGACCCAAGCAC	CAGGTCGTCATCATCCCACG
TNF	GACCCTCACACTCAGATCATCTTCT	TCCGCTTGGTTTGCTA
IL-10	CCTGGTAGAAGTGATGCCCC	TGTCACGTAGGCTTCTATGCAG
IL-6	CTGGTCTTCTGGAGTTCCGT	GGTCCTTAGCCACTCCTTCTG
GAPDH	CTGTTCTAGAGACAGCCGCATCT	ACACCGACCTTCACCATCTTG

9. Gut microbiota analysis

The fresh fecal samples in autoclaved sterile vials were properly packaged with dry ice cooling agents and shipped to BGI Genomics Co., Ltd (Hong Kong) for microbiota

analyses by sequencing the V4 region of the 16S ribosomal ribonucleic acid (rRNA) gene. Briefly, genomic DNA was extracted and used to generate 16S rRNA libraries using the Illumina MiSeq platform. After eliminating the adapter pollution and low quality within raw sequences, paired-end reads with overlap were merged to tags (minimal overlapping length: 15 bp; mismatching ratio of overlapped region: <= 0.1). Next tags were clustered to OTUs (Operational Taxonomic Units) by scripts of software USEARCH (v7.0.1090) with a 97% threshold. Taxonomic ranks were assigned to OTU representative sequence using Ribosomal Database Project (RDP) Naïve Bayesian Classifier v.2.2. At last, alpha diversity (Mothur, v1.31.2), beta diversity (QIIME, v1.80) and the different species screening were analyzed based on OTU and taxonomic ranks. Species clustering heat map was fabricated using the package 'gplots' of software R (v3.1.1). The distance algorithm is 'euclidean', and the clustering method is 'complete'.

Supplementary Figures

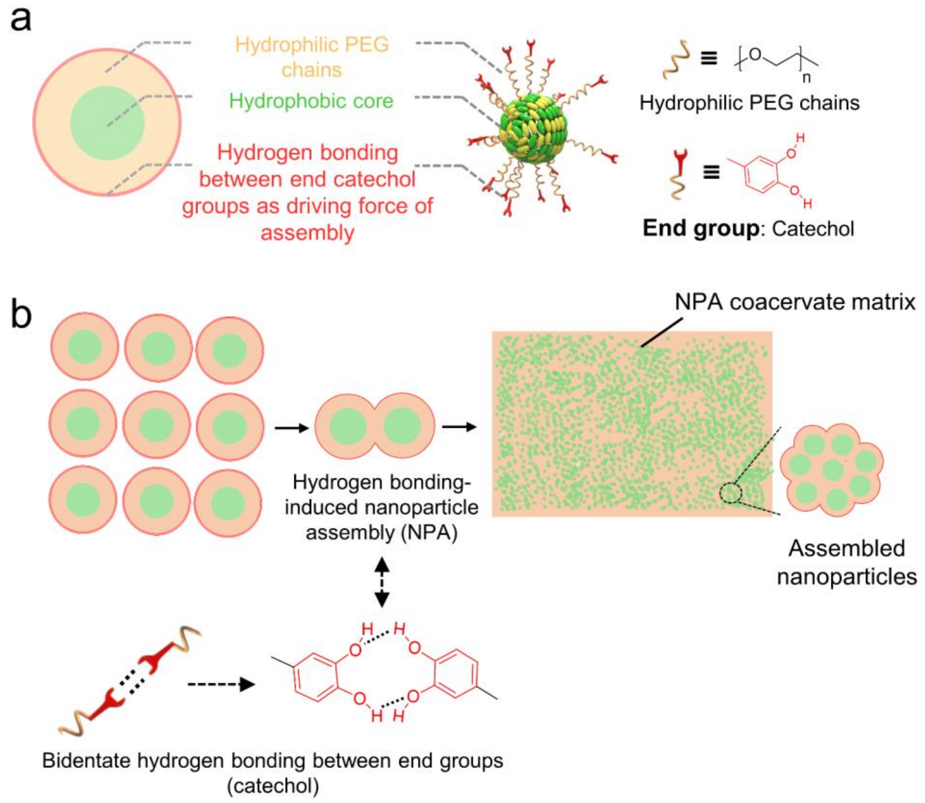

Supplementary Figure 1. a-b) The bidentate hydrogen bonding interactions between the two hydroxyl groups of catechol groups on the outer surface of NP2 nanoparticles induce the nanoparticle assembly to yield NPA coacervate.

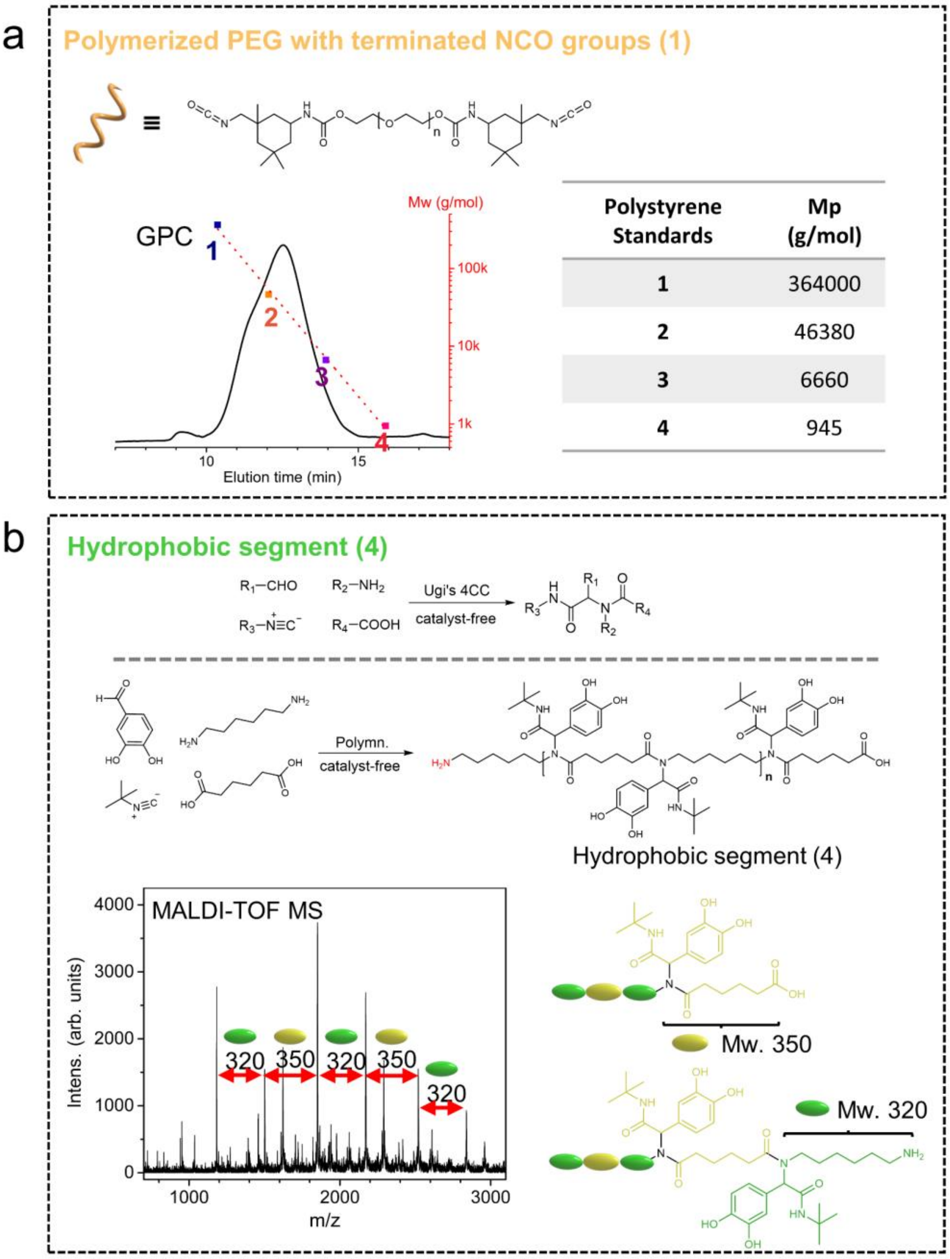

Supplementary Figure 2. a) The molecular weight distribution of the hydrophilic polymerized PEG chains was determined by gel permeation chromatography (GPC). b) MALDI-TOF mass spectrometry was used to confirm the successful synthesis of the hydrophobic segment (4) than can act as the hydrophobic core of nanoparticles.

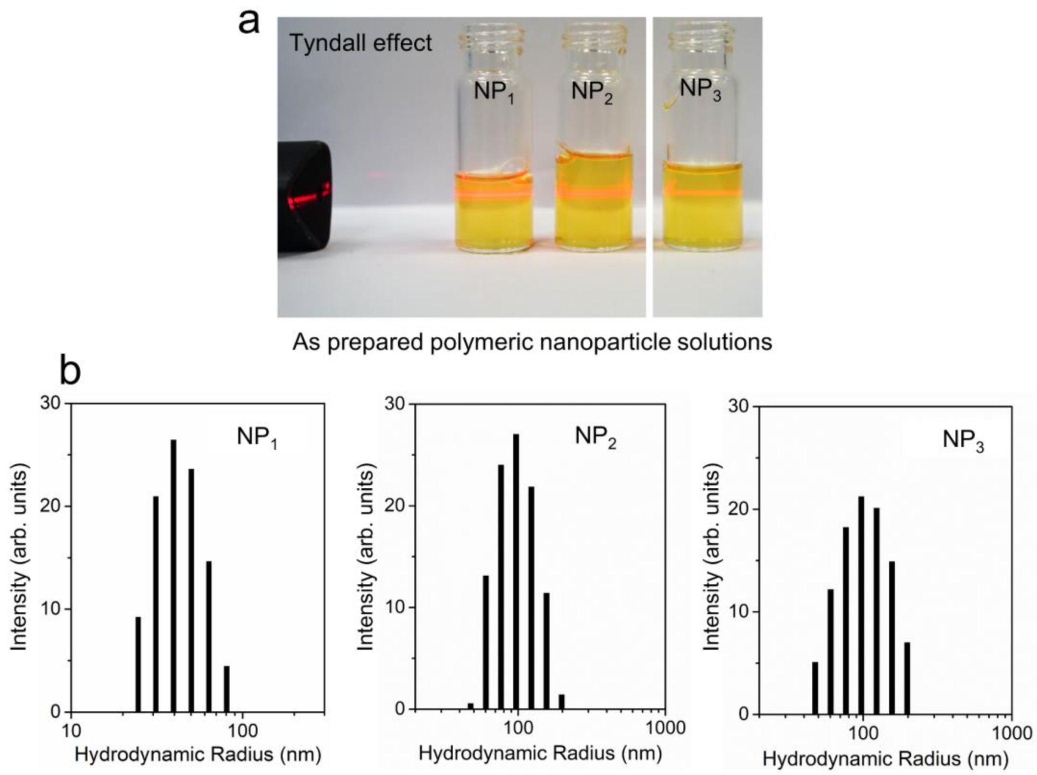

Supplementary Figure 3. a) The formation of as-prepared nanoparticles was observed by Tyndall effect. b) Dynamic light scattering (DLS) analysis confirmed the formation of the as-prepared NP_1, NP_2, and NP_3 nanoparticles.

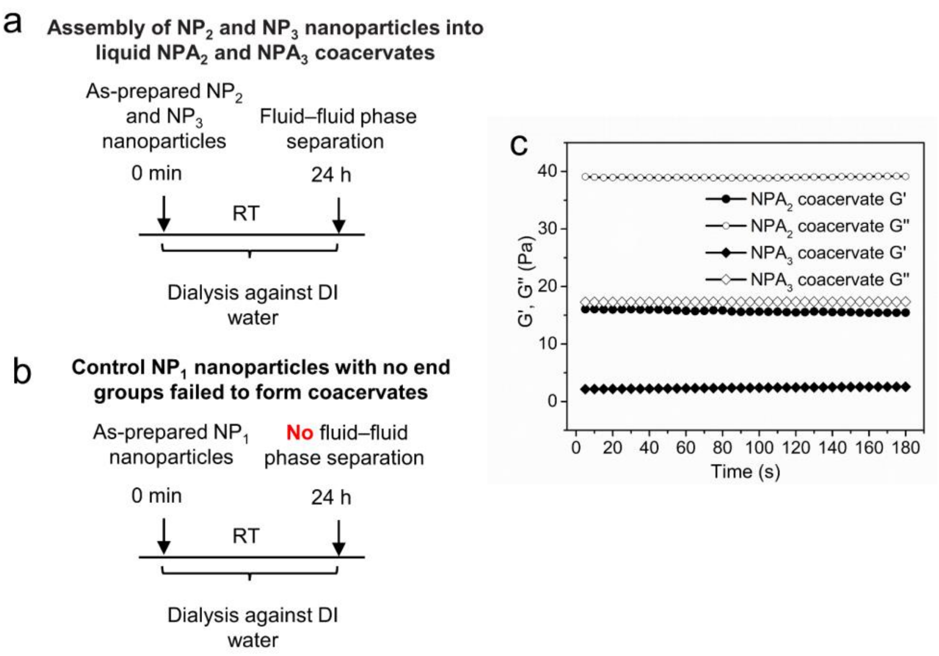

Supplementary Figure 4. a-c) The assembly of NP_2 and NP_3 nanoparticles formed of the liquid NPA_2 and NPA_3 coacervates (G’ < G”) confirmed via rheological time sweep. After 24 hours’ dialysis against DI water, the control NP_1 nanoparticles without surface end groups failed to form coacervate.

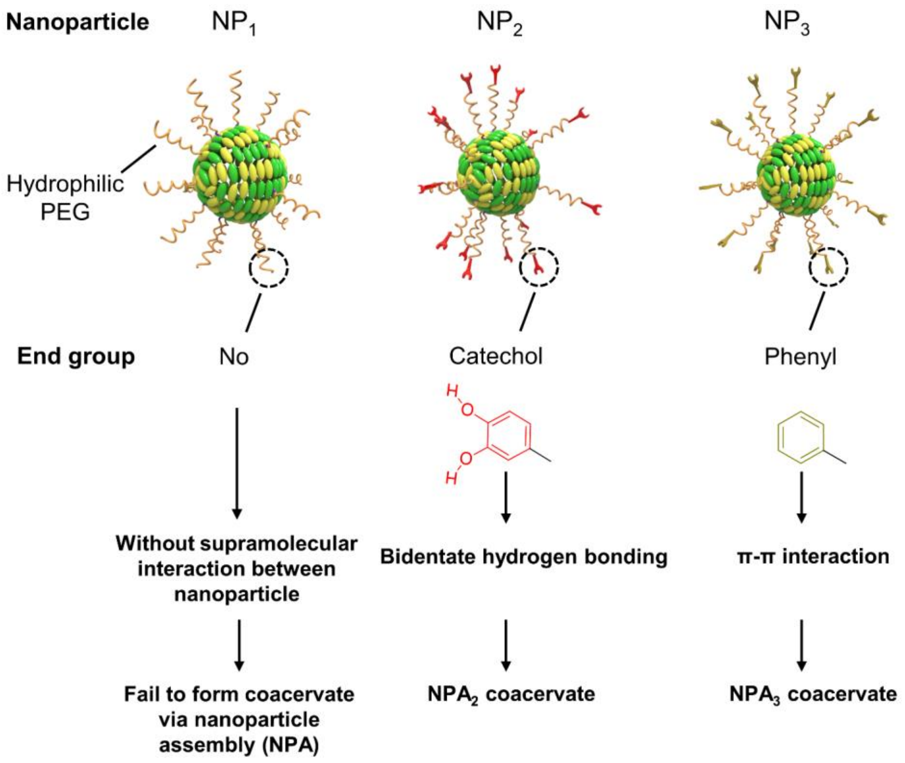

Supplementary Figure 5. Schematic illustration of the role of end groups (i.e., catechol, and phenyl) in facilitating nanoparticle assembly.

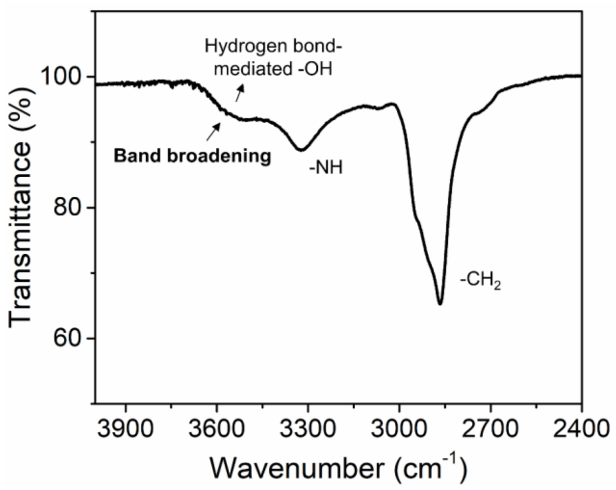

Supplementary Figure 6. FTIR result of freeze-dried NPA_2 coacervate showed obvious band broadening of phenolic hydroxyl groups (-OH) derived from catechol, thus confirming the existence of the hydrogen bonding interactions.

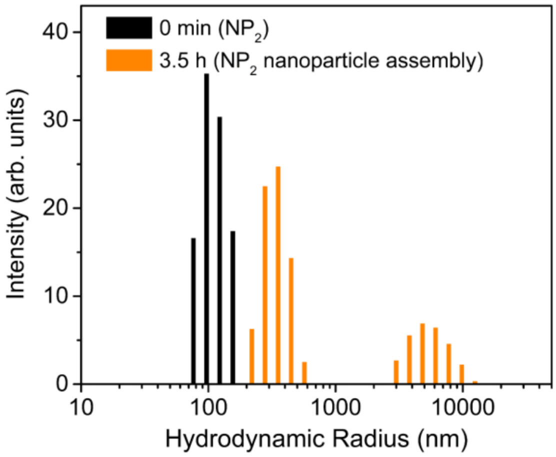

Supplementary Figure 7. DLS analysis showed assembly of NP_2 nanoparticles to form coacervate micro-droplets as confirmed by the increasing hydrodynamic radius of NP_2 nanoparticles after 3.5 hours of dialysis.

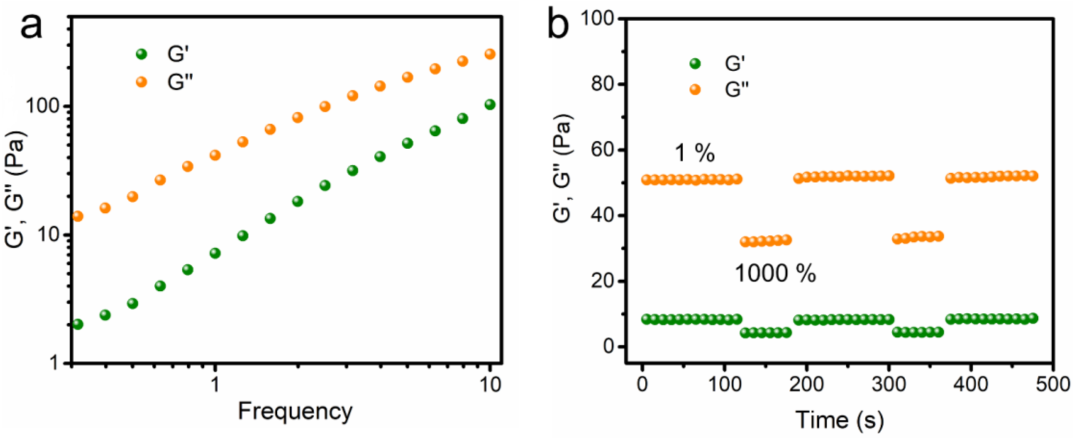

Supplementary Figure 8. a) Frequency-dependent storage (G') and loss (G'') of the liquid NPA_2 coacervate. b) Shear-thinning test revealed the inherent rapid self-healing ability of liquid NPA_2 coacervate.

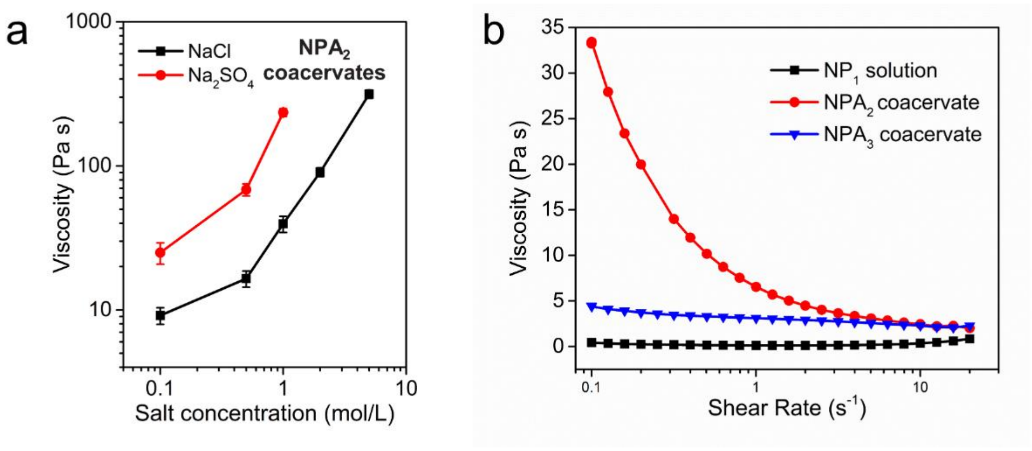

Supplementary Figure 9. a) Viscosity of NPA_2 coacervate increased with increasing salt concentrations. n = 2 independent samples per group. Data are presented as mean ± SD. b) The rheological analysis confirmed the shear-thinning behavior of the NPA coacervates. The viscosity of the NPA_2 coacervate decreased quickly with an increasing shear rate from 0.1 to 20.0 s^{-1}. In contrast, the NPA_3 coacervate demonstrated a much lower viscosity than that of the NPA_2 coacervate.

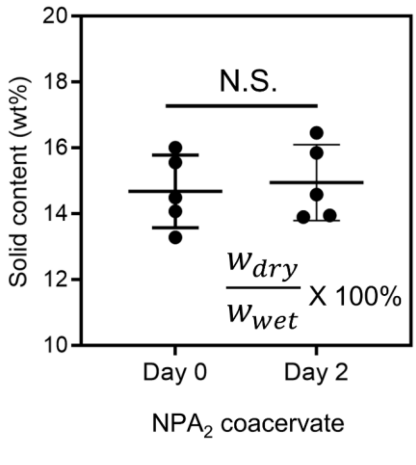

Supplementary Figure 10. The solid content of the non-swellable NPA_2 coacervate is around 15 wt%. n = 5 independent samples per group. Data are presented as mean ± SD. (N.S.) P > 0.05, *P < 0.05, **P < 0.01, ***P < 0.001 (two-tailed Student's t-test).

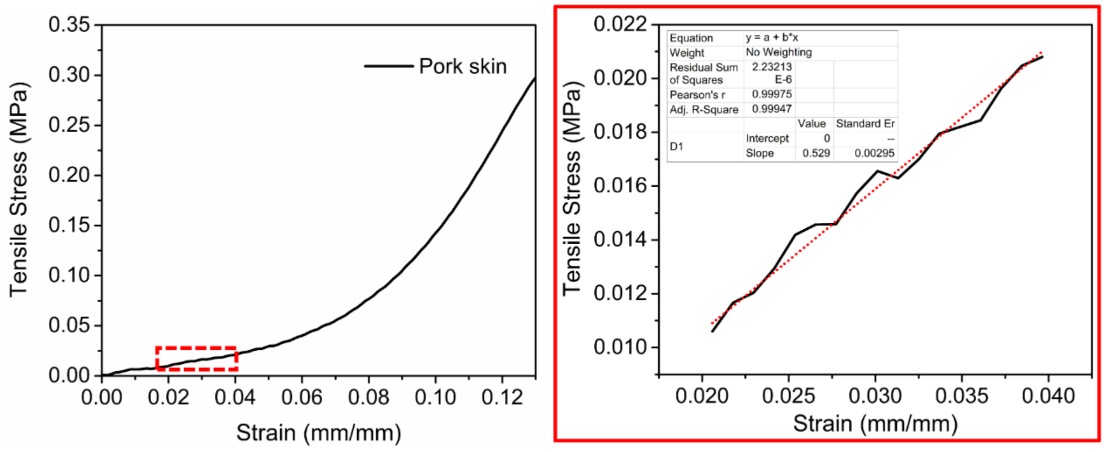

Supplementary Figure 11. Young's modulus of the fresh pork skin was calculated as the slope of the initial section of the stress-strain curve.

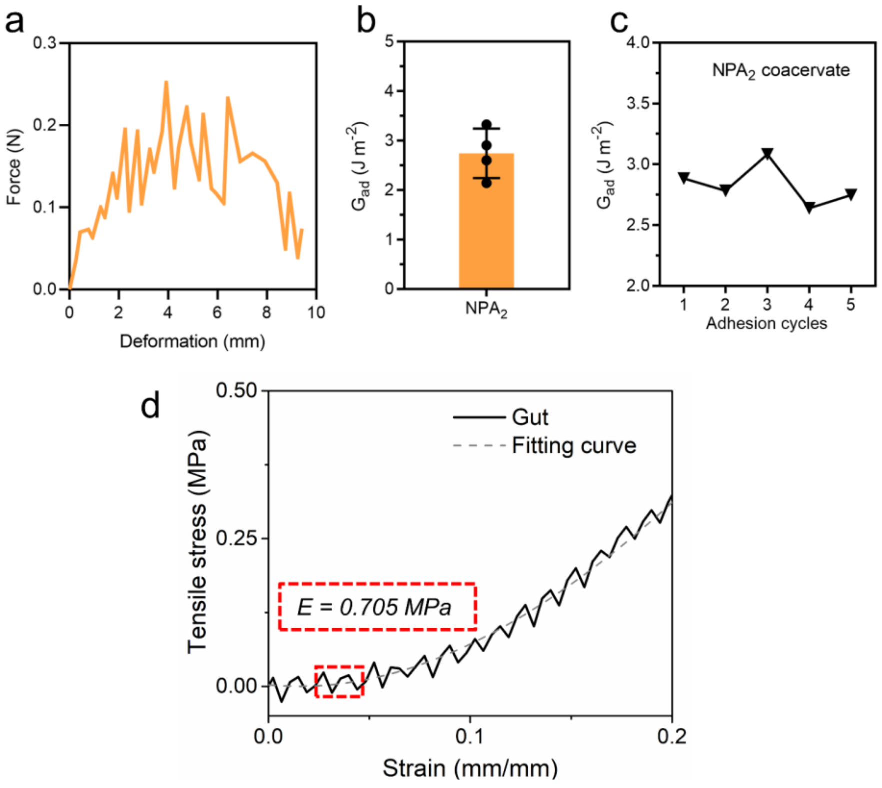

Supplementary Figure 12. a-c) NPA_2 coacervate showed reversible mucoadhesion on the luminal surface of porcine intestines. The adhesion energy was calculated by a method similar to the porcine skin lap shear test (Figure 3c) according to the protocol detailed in Supplementary Method 4: Lap shear adhesion measurement (Supporting Information)[4]. n = 4 independent lap shear tests for b). Data are presented as mean ±

SD. d) Young's modulus of the fresh porcine intestine was calculated as 0.705 MPa which is consistent with the previous report[9].

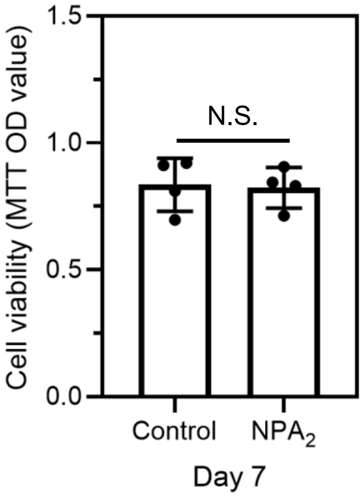

Supplementary Figure 13. Cell metabolic assays (MTT) of hMSCs incubated with NPA_2 coacervate for 7 days suggested excellent cytocompatibility of the NPA_2 coacervate. n = 4 independent experiments per group. Data are presented as mean ± SD. (N.S.) P > 0.05, *P < 0.05, **P < 0.01, ***P < 0.001 (two-tailed Student's t-test).

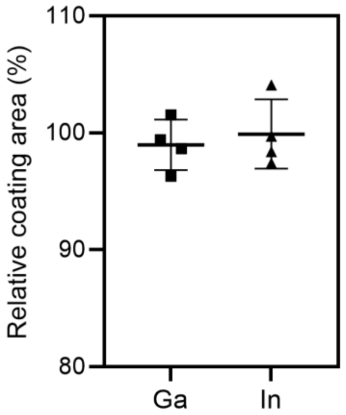

Supplementary Figure 14. After soaking in the simulated gastric fluid (Ga) and simulated intestinal fluid (In) at 37 °C for 2 hours, area of NPA_2 coacervate coating on

the fresh and wet mucosa remained unchanged compared to the coating area of NPA_2 coacervate before soaking. n = 4 independent experiments per group. Data are presented as mean ± SD.

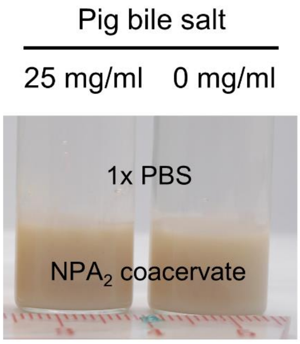

Supplementary Figure 15. NPA_2 coacervate remained largely stable in 25 mg/ml pig bile salt for at least 24 hours at 37 ℃.

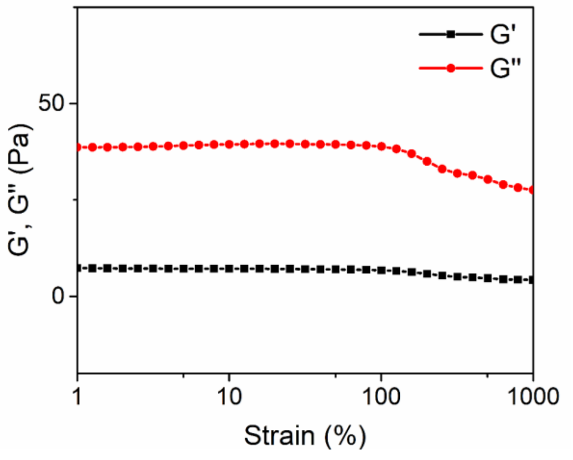

Supplementary Figure 16. The liquid-like (G' < G'') rheological properties of NPA_2 coacervate is stable under the high shear strain (up to 1000%).

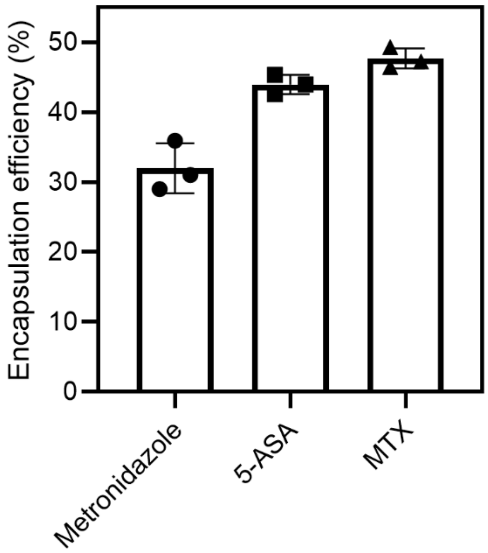

Supplementary Figure 17. Metronidazole (Metro), 5-ASA, and MTX were encapsulated into the NPA_2 coacervate with high encapsulation efficiency. n = 3 independent experiments per group. Data are presented as mean ± SD.

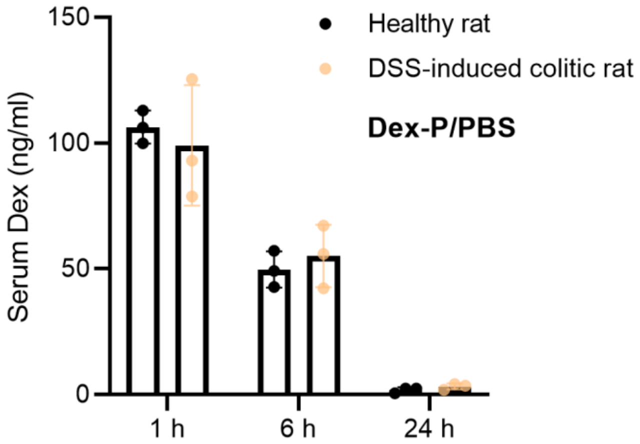

Supplementary Figure 18. Serum Dex concentration of DSS-induced colitic rats and healthy rats receiving the equivalent amount of Dex-P in PBS (Dex-P/PBS) were similar, indicating the similar in vivo Dex-P release kinetics between DSS-induced colitic rats and healthy rats in the absence of the coacervate carrier. n = 3 biologically

independent rats per group. Data are presented as mean ± SD.

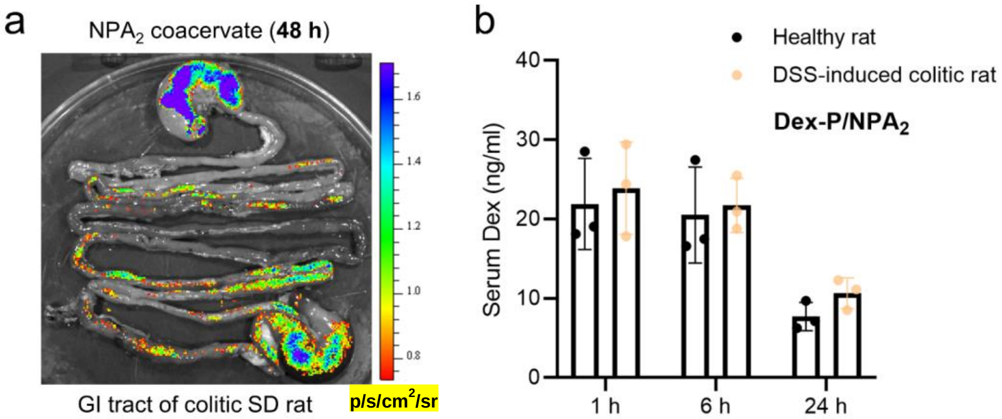

Supplementary Figure 19. a) The NPA$_2$ coacervate can adhere to the GI tract of colitic rats for at least 48 hours. b) Serum Dex concentration between DSS-induced colitic rats and healthy rats receiving an equivalent amount of Dex-P (Dex-P/NPA$_2$) were similar, indicating the similar in vivo Dex-P release kinetics between DSS-induced colitic rats and healthy rats in the presence of the coacervate carrier. n = 3 biologically independent rats per group. Data are presented as mean ± SD.

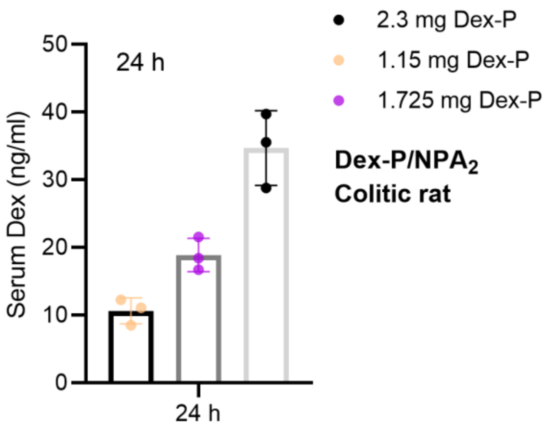

Supplementary Figure 20. Dose-dependent serum Dex concentration at 24 h in the

DSS-induced colitic rats receiving Dex-P/NPA_2. n = 3 biologically independent rats per group. Data are presented as mean ± SD.

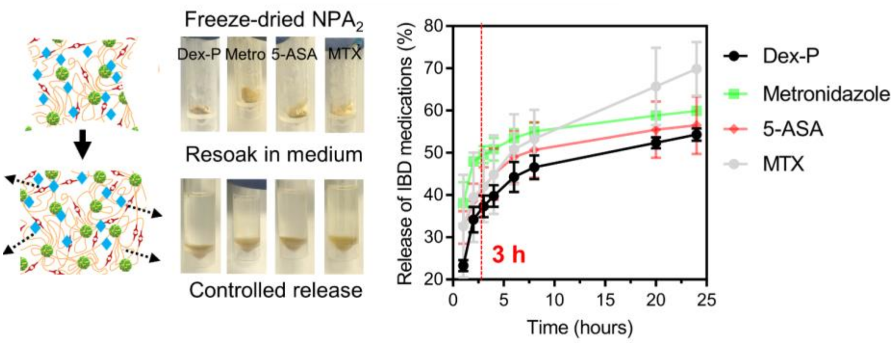

Supplementary Figure 21. The freeze-dried drug-laden NPA_2 coacervates could be easily rehydrated to fluid NPA_2 coacervate with release kinetics of diverse drugs similar to the freshly prepared drug-laden NPA_2 coacervate before lyophilization. n = 2 independent experiments per group. Data are presented as mean ± SD.

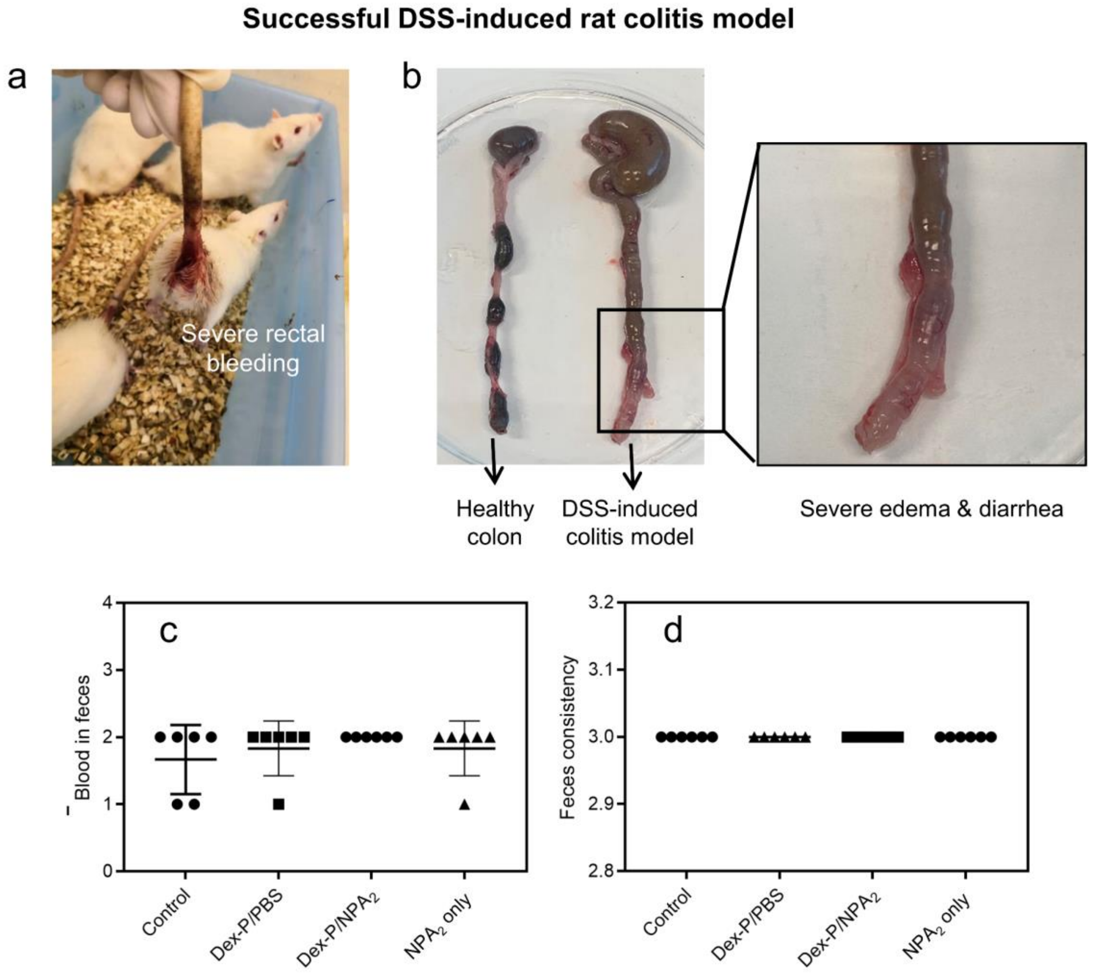

Supplementary Figure 22. a-b) Successful establishment of the colitis model in SD rats. Severe rectal bleeding, watery diarrhea, and colonic edema were observed after providing 4.5% DSS in drinking water to SD rats for 7 days. c-d) The colitic rats with similar c) feces consistency and d) blood in feces were then randomly assigned to the control, Dex-P/NPA_2, and Dex-P/PBS groups for the flowing treatment. n = 6 biologically independent rats per group. Data are presented as mean ± SD. Briefly, clinical scoring of the DSS-induced acute colitis was as follows[6]:

Feces consistency: 0, Normal; 1, wet; 2, soft; 3, Water diarrhea;

Blood in feces: 0, no blood; 1, bloody stools and/or blood around the anus; 2, severe bleeding;

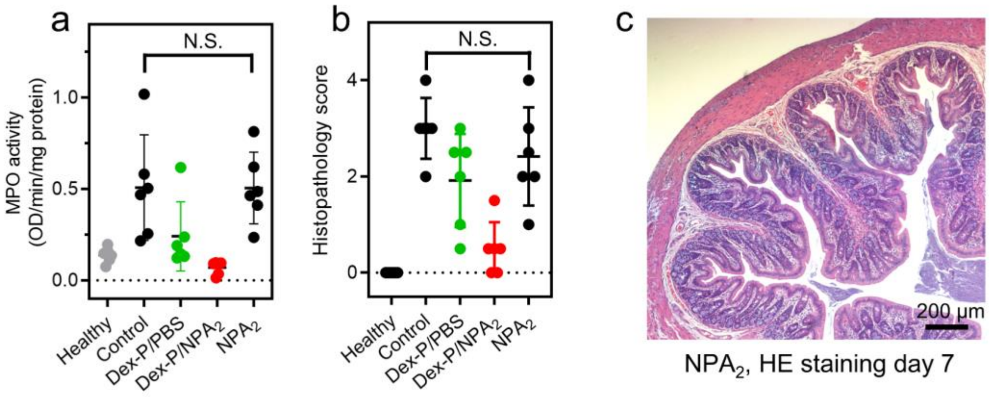

Supplementary Figure 23. The MPO activity, histopathology score, and HE staining indicate that the therapeutic efficacy of treatment with NPA_2 alone in colitic rats is not significantly different from that of the non-treated colitic rats (Control). n = 6 biologically independent rats per group. Data are presented as mean ± SD. (N.S.) P > 0.05, *P<0.05, **P<0.01, ***P<0.001 (Ordinary one-way ANOVA).

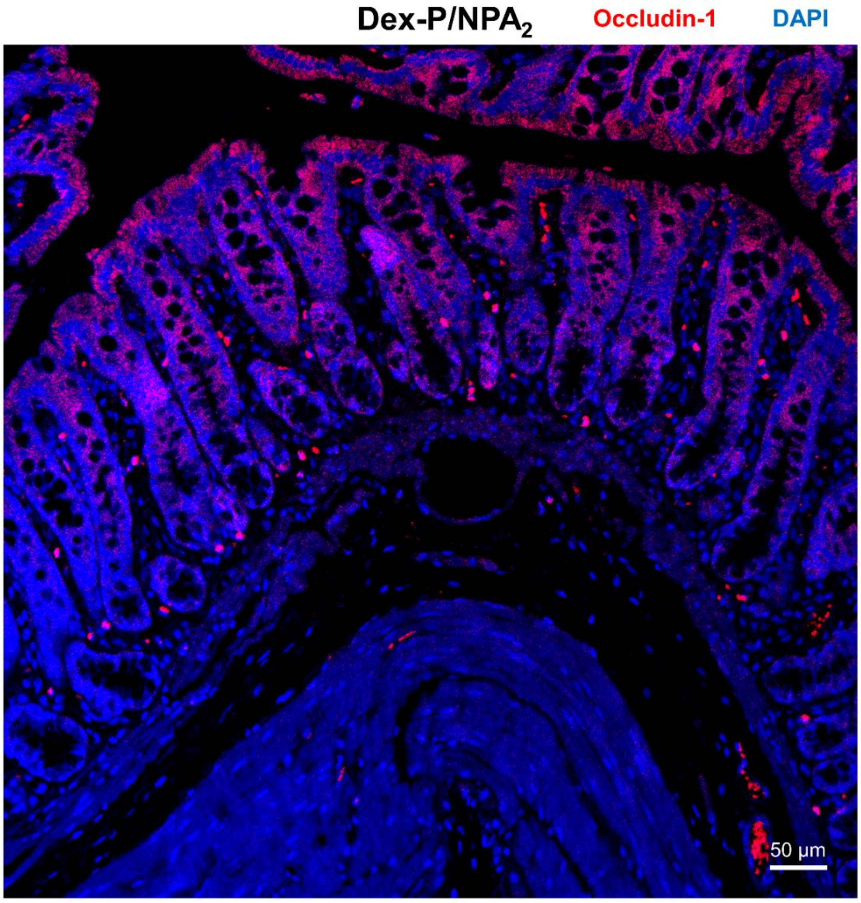

Supplementary Figure 24. Representative immunofluorescence staining against occludin-1 (red)/DAPI (blue) at low power field showed the overall staining status of the colonic section of colitic SD rats receiving Dex-P/NPA$_2$ at day 7. Similar results were found in the 6 biologically independent rats in Dex-P/NPA$_2$ group.

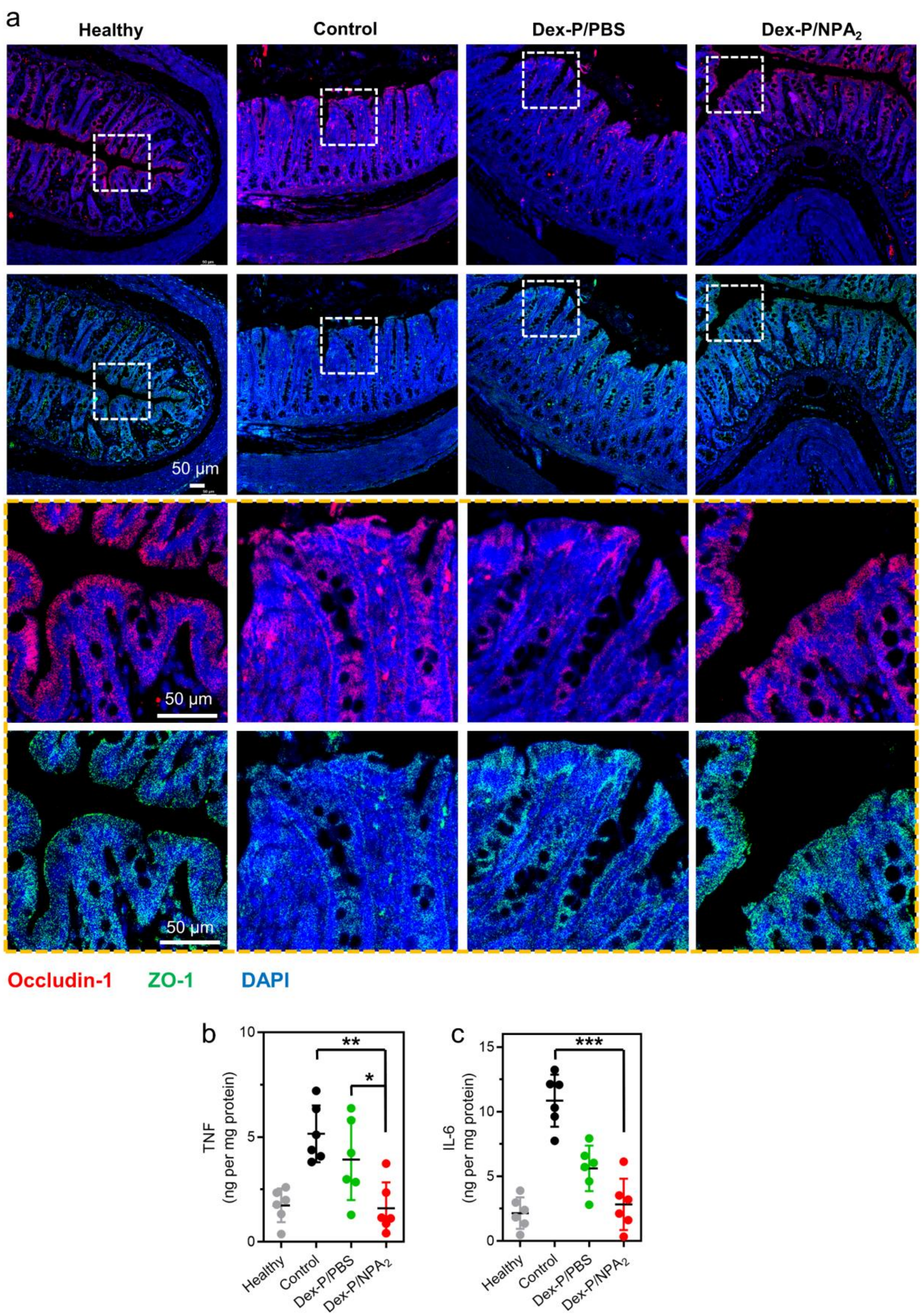

Supplementary Figure 25. Representative immunofluorescence staining images against ZO-1 (green) and occludin-1 (red) at high power field. a) On day 7, colon tissues

were harvested, stained with anti-Occludin-1 and anti-ZO-1 antibodies, and visualized by confocal microscopy. Colitic SD rats receiving Dex-P/NPA_2 recovered more expression of tight junction-associated proteins including ZO-1 and occludin-1, which continuously covered the colonic surfaces. b-c) Colon tissues were analyzed for the concentrations of pro-inflammatory cytokines including TNF and IL-6 via ELISA. n = 6 biologically independent rats per group. Data are presented as mean ± SD. *P<0.05, **P<0.01, ***P<0.001 (Ordinary one-way ANOVA).

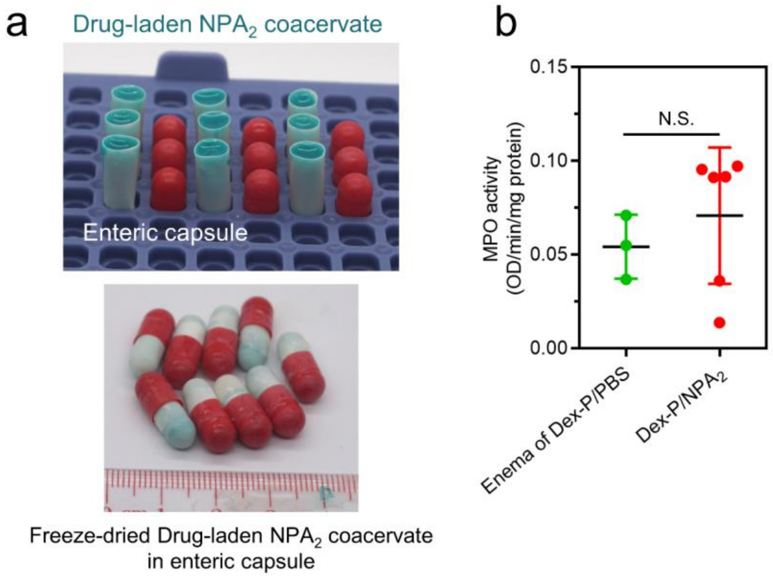

Supplementary Figure 26. a) Drug-laden NPA_2 coacervate can be encapsulated into the enteric capsules, thus potentially combining both the advantages of sustained release and targeting of the desired region. b) Treatment with Dex-P/PBS via enema on days 1, 3, and 5 decreased the colonic MPO activity in colitic SD rats at day 7 to a similar level as that of the oral delivery of Dex-P/NPA_2. n = 3 biologically independent rats in Enema of Dex-P/PBS group, n = 6 biologically independent rats in Dex-P/NPA_2 group. Data are presented as mean ± SD. (N.S.) P > 0.05, *P < 0.05, **P < 0.01, ***P < 0.001 (two-tailed Student's t-test).

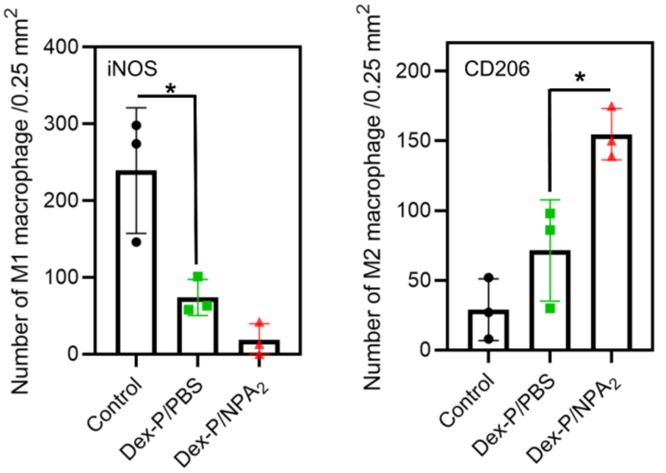

Supplementary Figure 27. Quantification of macrophages positive of M1 (iNOS) and M2 (CD206) markers indicates that oral administration of Dex-P/NPA$_2$ to colitic SD rats promoted anti-inflammatory M2 macrophage polarization (CD206) and suppressed pro-inflammatory M1 polarization (iNOS). n = 3 randomly selected immunohistochemical images from 5 biologically independent rats per group. Data are presented as mean ± SD. *$P<0.05$, **$P<0.01$, ***$P<0.001$ (Ordinary one-way ANOVA).

Supplementary References

1. Sehlinger A, Dannecker P-K, Kreye O, Meier MA. Diversely substituted polyamides: macromolecular design using the Ugi four-component reaction. *Macromolecules* **47**, 2774-2783 (2014).
2. Dömling A, Ugi I. Multicomponent reactions with isocyanides. *Angew Chem Int Ed* **39**, 3168-3210 (2000).
3. Zhao P*, et al.* Mussel-mimetic hydrogels with defined cross-linkers achieved via controlled catechol dimerization exhibiting tough adhesion for wet biological tissues. *Chem Commun* **53**, 12000-12003 (2017).
4. Rose S, Prevoteau A, Elzière P, Hourdet D, Marcellan A, Leibler L. Nanoparticle solutions as adhesives for gels and biological tissues. *Nature* **505**, 382 (2014).
5. Seo S*, et al.* Microphase behavior and enhanced wet-cohesion of synthetic copolyampholytes inspired by a mussel foot protein. *J Am Chem Soc* **137**, 9214-9217 (2015).
6. Martin JC, Bériou G, Josien R. Dextran sulfate sodium (DSS)-induced acute colitis in the rat. In: *Suppression and Regulation of Immune Responses*). Springer (2016).
7. Wilson DS, Dalmasso G, Wang L, Sitaraman SV, Merlin D, Murthy N. Orally

delivered thioketal nanoparticles loaded with TNF-α–siRNA target inflammation and inhibit gene expression in the intestines. *Nat Mater* **9**, 923-928 (2010).

8. Zhang S*, et al.* An inflammation-targeting hydrogel for local drug delivery in inflammatory bowel disease. *Sci Transl Med* **7**, 300ra128-300ra128 (2015).
9. Guimarães CF, Gasperini L, Marques AP, Reis RL. The stiffness of living tissues and its implications for tissue engineering. *Nature Reviews Materials* **5**, 351-370 (2020).
